# Chinook salmon depth distributions on the continental shelf are shaped by interactions between location, season, and individual condition

**DOI:** 10.1186/s40462-024-00464-y

**Published:** 2024-03-15

**Authors:** Cameron Freshwater, Sean C. Anderson, David D. Huff, Joseph M. Smith, Doug Jackson, Brian Hendriks, Scott G. Hinch, Stephen Johnston, Andrew W. Trites, Jackie King

**Affiliations:** 1 Pacific Biological Station, Fisheries and Oceans Canada, Nanaimo, BC Canada; 2 Northwest Fisheries Science Center, National Marine Fisheries Service, Seattle, WA USA; 3 QEDA Consulting, Seattle, WA USA; 4 Pacific Salmon Ecology and Conservation Laboratory, Department of Forest and Conservation Sciences, The University of British Columbia, Vancouver, BC Canada; 5 Marine Mammal Research Unit, Institute for the Oceans and Fisheries, The University of British Columbia, Vancouver, BC Canada

**Keywords:** Habitat use, Telemetry, Seasonality, ROMS, Bathymetry, Machine learning models

## Abstract

**Background:**

Ecological and physical conditions vary with depth in aquatic ecosystems, resulting in gradients of habitat suitability. Although variation in vertical distributions among individuals provides evidence of habitat selection, it has been challenging to disentangle how processes at multiple spatio-temporal scales shape behaviour.

**Methods:**

We collected thousands of observations of depth from $>~300$ acoustically tagged adult Chinook salmon *Oncorhynchus tshawytscha*, spanning multiple seasons and years. We used these data to parameterize a machine-learning model to disentangle the influence of spatial, temporal, and dynamic oceanographic variables while accounting for differences in individual condition and maturation stage.

**Results:**

The top performing machine learning model used bathymetric depth ratio (i.e., individual depth relative to seafloor depth) as a response. We found that bathymetry, season, maturation stage, and spatial location most strongly influenced Chinook salmon depth. Chinook salmon bathymetric depth ratios were deepest in shallow water, during winter, and for immature individuals. We also identified non-linear interactions among covariates, resulting in spatially-varying effects of zooplankton concentration, lunar cycle, temperature and oxygen concentration.

**Conclusions:**

Our results suggest Chinook salmon vertical habitat use is a function of ecological interactions, not physiological constraints. Temporal and spatial variation in depth distributions could be used to guide management decisions intended to reduce fishery impacts on Chinook salmon. More generally, our findings demonstrate how complex interactions among bathymetry, seasonality, location, and life history stage regulate vertical habitat selection.

**Supplementary Information:**

The online version contains supplementary material available at 10.1186/s40462-024-00464-y.

## Introduction

Species exhibit variation in habitat use across spatial and ecological scales. In the case of mobile organisms, habitat use also varies temporally, and individual movement patterns can determine survival and reproductive success [1]. Movement is commonly used to identify processes regulating species distributions, which in turn can improve predictions of how populations will respond to future stressors. For instance, polar bear *Ursus maritimus* habitat use is moderated by sea ice availability [2], while the migratory patterns of elk *Cervus elaphus* are regulated by interactions between vegetation phenology, quality, and abundance [3].

In the case of aquatic species, however, habitat selection occurs along both horizontal and vertical dimensions. Indeed, environmental gradients often vary more rapidly with depth than with horizontal distance, resulting in discrete species distributions [4]. For example, capelin *Mallotus villosus* are physiologically constrained by subzero temperatures to specific depths in the North Atlantic [5]. In other cases, vertical habitat use covaries with ecological interactions. Tagging data indicate African penguin *Spheniscus demersus* foraging success varies between benthic and pelagic habitats [6]. The depth distributions of large pelagic fishes are shaped by interactions between both physiological and ecological processes. Species with suitable physiological adaptations are able to exploit cold, productive waters hundreds of meters below the surface, while others are limited to foraging in surface waters [7]. The changing climate will likely restructure vertical habitats’ ecological characteristics [8, 9]. Thus, understanding the processes shaping depth distributions is necessary to predict how aquatic populations respond to changing environmental conditions.

An improved understanding of how populations use vertical habitats can also directly inform management. Variation in depth can influence a population’s vulnerability to fisheries [10] and have been used to tailor fisheries restrictions to minimize impacts on non-target species (e.g., depth-based rockfish conservation areas [11]). Similarly, catchability effects associated with depth can bias indices of abundance and may require modified assessment methods [12]. Fisheries are increasingly regulated by ecosystem-based management, which necessitates an improved understanding of species interactions. Overlapping distributions in both vertical and horizontal space can be used to identify plausible trophic relationships [13, 14].

Unfortunately the ability to predict vertical habitat use is challenged by the diverse processes that influence depth distributions. First, vertical movements consistently differ among habitat types, resulting in patterns across horizontal space [15]. Bottom depth constrains the vertical habitat accessible to an individual, and complex bottom topographies often serve to aggregate prey thereby impacting predator behaviour [16, 17]. Residual spatial variation in depth distributions may represent physical oceanographic processes, such as offshore currents, which are difficult to observe, but form discrete habitat structures. Second, vertical movements can vary with time of day and year. Diel vertical migrations (DVM) are particularly widespread [18]. At intermediate temporal scales, changing tides or lunar illumination may moderate ecological interactions [7, 16]. Seasonal changes in depth distributions may also arise due to physiological constraints, changes in the relative abundance of different prey, or life history events such as reproduction [19–21]. Third, vertical distribution may consistently differ among individuals. Depth distributions may change as fish reach reproductive maturity [22, 23]. Individuals also often select specific habitats to balance predation risk and foraging requirements [24, 25]. Thus, individuals in poor condition or at greater risk of predation may show distinct vertical distributions.

Importantly, spatial, temporal, and individual processes can interact with one another so that vertical movements are most pronounced under specific conditions. The relative frequency of DVM is moderated by predator communities, prey availability, and individual condition [18]. Blue sharks use ephemeral mesoscale eddies to expand their thermal niche into mesopelagic ecosystems [26]. Maturity stage and habitat influence the vertical distributions of anadromous salmonids—individuals exploit surface waters for olfactory cues in nearshore marine habitats, but use deep water thermal refugia during freshwater migrations [27–29].

Chinook salmon *Oncorhynchus tshawytscha* is a piscivorous, anadromous species found in pelagic and neritic habitats in the North Pacific. Chinook salmon support fisheries throughout the region and provide unique ecosystem services, including serving as prey for resident killer whales *Orcinus orca* [30]. A more nuanced understanding of Chinook salmon habitat use can provide novel insights into their ecology and guide management actions. For example, current spatio-temporal closures reflect the two-dimensional habitat use of salmon. Vertical distributions could be used to highlight areas where interactions between Chinook salmon, predators, and prey are most common [13]. Similarly, depth data could be used to minimize interactions between Chinook salmon and midwater trawl fisheries that intercept salmon as bycatch [31, 32]. Typically Chinook salmon occupy depths from the surface to several hundred meters, and variation in vertical habitat use has been attributed to ontogeny, environmental conditions, diel cycles, season, and geographic location [32–38]. Yet, previous studies have not considered these varied processes simultaneously.

**Fig. 1:**
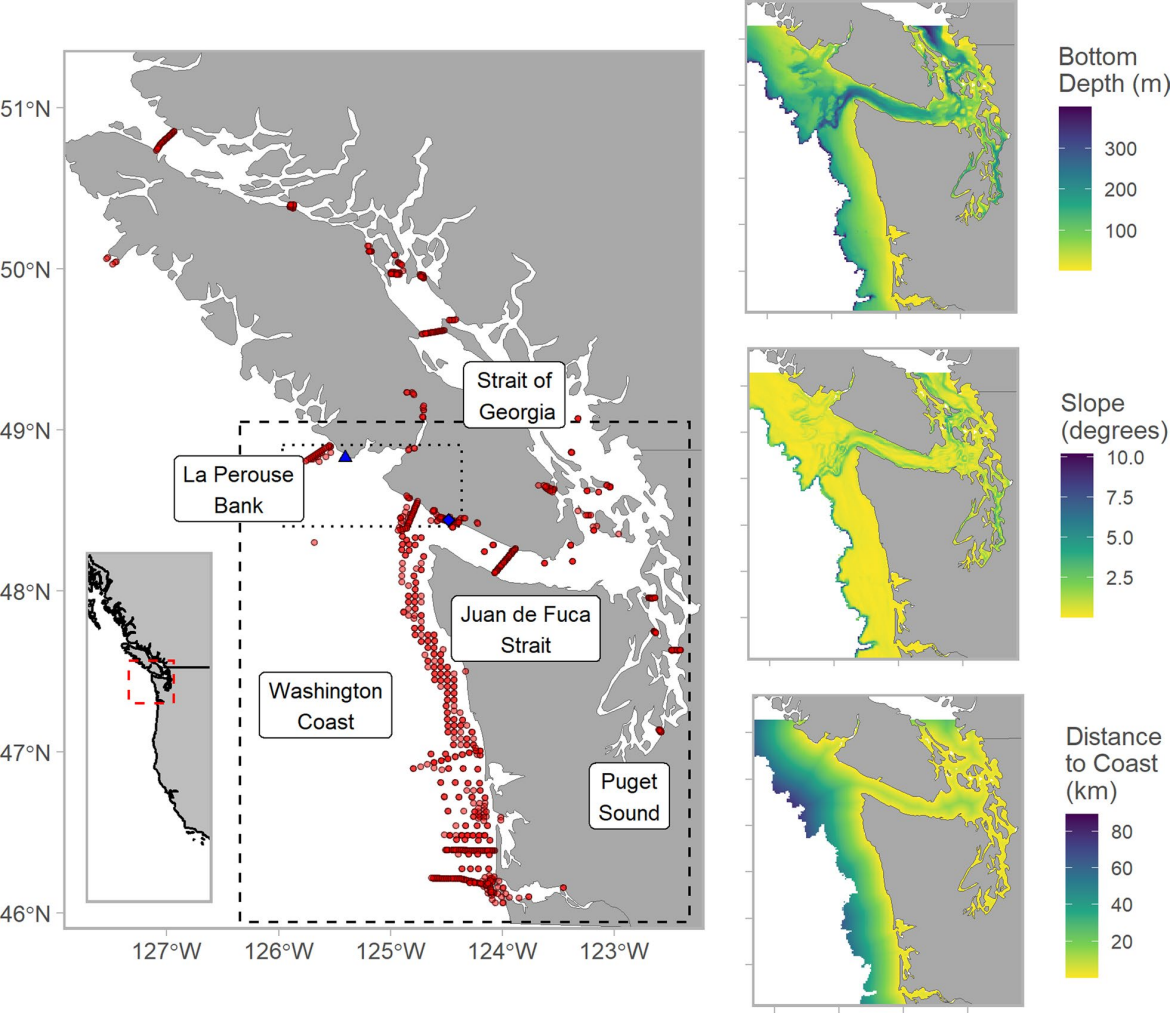
Study area including receiver locations (red circles), as well as locations where Chinook salmon were tagged—Ucluelet (blue triangle) and Port Renfrew (blue diamond). Major geographic features are labelled. Note that the Salish Sea includes Juan de Fuca Strait, the Strait of Georgia, and Puget Sound. The approximate location of tag releases and the focal study area where model predictions were made are shown as rectangles (dotted and dashed respectively). Detailed tag release locations are shown in Fig S1. Location of study area relative to west coast of North America is shown by inset with focal study area represented by dashed box. Column on right shows spatial variables within the study area inset: bottom depth (top), slope (middle) and distance to nearest coastline (right). Projection is UTM Zone 10

Disentangling the relative influence of multiple processes on vertical movements requires observations that span diverse horizontal habitats, multiple seasons, and a large number of individuals. Here we use observations of more than 300 adult Chinook salmon tagged with long-lived depth-sensing acoustic transmitters. Individual fish were detected using multiple acoustic receivers arrays from the west coast of Vancouver Island (WCVI) to the mouth of the Columbia River and throughout the Salish Sea (Fig. 1). We incorporated an array of explanatory variables that spanned spatial, temporal, and biological processes, including dynamic oceanographic variables from a local Regional Ocean Modeling System (ROMS). We then used a machine learning model to evaluate the relative predictive power of different covariates. Ultimately, this framework allowed us to estimate additive effects among covariates, such as how the effect of temporal and biological covariates differed through space. Our analysis provides inference on the primary processes shaping vertical habitat use in a commercially, culturally, and ecologically important aquatic species.

## Methods

### Study species and area

Chinook salmon are anadromous, semelparous, and, relative to other species of Pacific salmon, have diverse life-history strategies [39]. During marine life stages, Chinook salmon become increasingly piscivorous, feeding on forage fish and squid [39, 40]. We collected observations of Chinook salmon vertical habitat use from northern Vancouver Island to the Columbia River estuary, with the majority of data collected near central Vancouver Island, the coast of southern Washington, Juan de Fuca Strait, and Puget Sound. The study region is bathymetrically diverse, including extensive continental shelf habitats, networks of canyons, narrow straits, and shallow inland seas (Fig. 1). Many distinct Chinook salmon populations, originating from central California to northern British Columbia, can be encountered in this region [41, 42]. Some populations use the continental shelf near WCVI and the Salish Sea (defined as Juan de Fuca Strait, the Strait of Georgia, Puget Sound, and their adjoining waterways) as year-round rearing habitats, while others use the region seasonally as a migratory corridor [42]. We note, however, that our study does not fully represent Chinook salmon life-history diversity since early run timing, yearling populations were rarely encountered and our study area encompasses only a fraction of the marine distribution of a species that spawns as far north as Norton Sound, as well as in Asia [39].

### Tag deployments and receiver arrays

Two field programs tagged Chinook salmon. From 2019 to 2022, fish were sampled by Fisheries and Oceans Canada (DFO) near Ucluelet, British Columbia, using hook-and-line gear fished from a commercial troller. Sampling took place between late April and early September. Fish that were landed without major injuries (e.g., eye damage, heavy bleeding; n = 312) received coded acoustic transmitters (Innovasea Inc.; model V13P; 39 mm length, 5.5 g weight in water, approximately 400 days battery life; mean 120-second delay between transmissions at 69 kHz). Tagging occurred on board the vessel. We transferred fish to a tagging sling with continuous flow-through of ambient seawater. We measured each fish using a tape (fork length and girth), removed a sample from the adipose or caudal fin for genetic stock identification, inserted a passive integrated transponder (PIT) tag, attached the acoustic transmitter, and estimated each individual’s energy density using a microwave oscillator, then immediately released the fish. Total handling time never exceeded six minutes. We used Biomark APT12 FDX-B PIT tags and estimated condition using a Distell Model 692 Fish Fatmeter, which provides a non-invasive index of lipid content [43]. We mounted acoustic transmitters externally via a Floy spaghetti tag fixed through the musculature posterior to the dorsal fin. Stock identities were assigned when individual assignment probabilities, estimated using single nucleotide polymorphisms [44], exceeded 80% within a given stock aggregate (Additional file 2: Table S1).

A second, University of British Columbia field program deployed acoustic transmitters in 2019 and 2020 (June-August) near Port Renfrew, British Columbia. Fish were sampled using a recreational vessel and sport fishing gear (n = 149), however, the tagging protocol was nearly identical to the DFO protocol, except fish did not receive a PIT tag. Floy spaghetti tags for both DFO and UBC programs included contact information in case a tagged fish was recovered by anglers, hatchery personnel, or biologists conducting spawner surveys. Fish were released between La Perouse Bank and Juan de Fuca Strait (Additional file 3: Fig. S1).

We detected tagged fish on Innovasea acoustic receivers (VR2, VR3, and VR4 models) that were deployed throughout the study area. These arrays were managed by a diverse network of scientists and the configuration of the arrays differed among years (Additional file 3: Fig. S7). Details in Online Supplement.

### Explanatory variables

We quantified relationships between depth and explanatory variables associated with detections (spatial location, time, oceanography) or the characteristics of individual fish at tagging (Table 1). We also considered a model that included stock aggregate identity as an additional covariate (Additional file 2: Table S1).

**Table 1:** Explanatory variables included in statistical analysis. Names in the second column match Fig. 3

Group	Covariate	Definition
Spatial	Location (UTM X and Y)	Easting and northing of receiver in UTM zone 10
	Shoreline distance	Distance (km) to coastline
	Bottom depth	Mean depth (m) of seafloor
	Bottom slope	Mean slope ($^{\circ }$) of seafloor
Temporal	Year Day (1 and 2)	Calendar day converted to two variables representing cyclical process
	Lunar cycle	Proportion of moon illuminated (0-1)
	Day-night	Whether detection occurred after sunrise and before sunset or not
Oceanographic	Temperature	Sea surface temperature ($\hbox {C}^{\circ }$)
	Hor. Current 1	Horizontal momentum (m/s) in N-S direction
	Hor. Current 2	Horizontal momentum (m/s) in E-W direction
	Vert. Current	Vertical momentum (m/s)
	Zooplankton	Zooplankton concentration ($\frac{\text { mmol N} }{\text { m }^3})$
	Oxygen	Dissolved oxygen concentration ($\frac{\text { mmol O} }{\text { m }^3})$
	Thermocline depth	Mixed layer depth (m) based on vertical temperature profiles
Individual	Size	Fork length when tagged (cm)
	Lipid content	Converted whole body lipid content (% wet weight)
	Maturity	Probability that an individual will mature in the same year as detection event

#### Spatial variables

We included the location of each detection (UTM coordinates in zone 10) and the distance to the nearest shoreline. We also included two bathymetric variables—bottom depth and bottom slope. Since the precise location of a detection within a receiver’s detection radius is unknown, we calculated each receiver’s mean bottom depth and mean slope within an 800 m radius. Bathymetric data were downloaded from NOAA’s 3-arc second (British Columbia; [45]) or 1/3-arc second (coastal Washington; [46]) digital elevation models. By including spatial coordinates as covariates, we developed a model that accounted for residual spatial variation that was present after spatially correlated variables were incorporated.

#### Temporal variables

We included calendar day of detection, which was transformed to represent seasonal effects (details in Online Supplement). To account for lunar effects, we calculated the proportion of the moon’s face that was illuminated at a given date and geographic location using the oce R package [47]. To account for diurnal effects, we created a categorical variable representing whether a detection event occurred during or after daylight hours (defined as sunrise and sunset), based on local time and the spatial location of the detection, using the suncalc R package [48].

#### Oceanographic variables

We obtained covariates representing dynamic oceanographic conditions from the LiveOcean configuration [49] of the Regional Ocean Modeling System (ROMS) [50, 51]. ROMS is a free-surface, primitive equations ocean circulation model that simulates ocean responses to physical forcing by wind, heat, tides, and other drivers. All LiveOcean variables were extracted at the surface (preliminary results were qualitatively similar with 25 m depth). We calculated thermocline depth using the rLakeAnalyzer R package [52], based on vertical profiles of temperature extracted at each detection location. We used temperature and oxygen concentration estimates at depth for each detection to visualize habitat use. Details in Online Supplement.

#### Individual variables

We included individual measurements of fork length and an index of lipid content to account for differences in individual condition at the time of tagging. We used a Distell Model 692 Fish Fatmeter (Distell Inc., West Lothian, Scotland), to generate indices of tissue lipid concentration. The Fatmeter uses a microwave oscillator to emit a low-powered wave that interacts with water in the somatic tissues at a given body location. We took Fatmeter readings at two positions anterior of the dorsal fine and above the lateral line [43]. We averaged these data to generate a single index, which was converted to an estimate of whole body lipid content following methods outlined in Lerner and Hunt [53].

Unlike most Pacific salmon, many populations of Chinook salmon mature in coastal environments along the continental shelf. Additionally, since Chinook salmon mature at multiple ages [39], immature and mature individuals are sympatric in our study area. We wanted to test for maturation stage effects since individuals that will remain at sea for at least one more year may be less likely to exhibit directed migratory behaviour. Unfortunately, we could not infer maturity stage at time of capture because Chinook salmon did not show external signs of sexual maturity. Therefore we assigned maturity stage post-hoc in a two-step process. In the first step, all fish detected in-river (via acoustic telemetry, PIT arrays, harvest, escapement walks, or hatchery broodstock removals) in the year of tagging were classified as mature (stage fixed at one), and all fish that were detected on marine arrays after November 15th in the year of tagging were classified as immature (stage fixed at zero). In the second step, we estimated the probability of maturation for the remaining individuals (approximately one-third of the total) based on size at the time of capture, capture date, and stock identity. Briefly, we fit a logistic regression model to fish of known maturity stage, including fork length and capture date as fixed effects and a random intercept for stock identity. We used the fitted model to generate individual-level posterior median maturation probabilities ranging from zero (immature) to one (mature). We then included maturation probability as a continuous explanatory variable, propagating uncertainty in maturation stage. Immature fish were considered mature on May 1 of the year following tagging. Details in Online Supplement.

### Statistical modeling

Depth data collected from acoustic transmitters have several characteristics that complicate analyses that use likelihood-based models, which depend upon an underlying statistical distribution. First, detection data are serially autocorrelated with substantial temporal and spatial structure. Second, depth is not normally distributed but bounded by zero and, in this case, right skewed towards surface depths. Third, depth distributions are constrained by bottom depth, resulting in a truncated distribution that varies spatially. We explored hierarchical generalized additive models with autoregressive residuals that can account for several of these issues, but found that they either failed to converge, showed significant spatial and temporal autocorrelation in their residuals, or required substantial data preprocessing to meet diagnostic checks (additional details below and in Online Supplement).

As an alternative, we used machine learning algorithms to estimate the relative importance of covariates on detection depth, estimate non-linear and interactive effects on detection depth, and generate spatially explicit predictions. Machine learning models do not assume the response variable follows an underlying statistical distribution, increasing flexibility when modeling data with a non-standard truncated distribution. Here we describe one algorithm, random forest regression [54], but we also evaluated gradient boosting machines ([55]; see Online Supplement for details). Random forest regression algorithms are built on regression trees. Each regression tree splits observations along a covariate at the point minimizing the sum of squares error, where the prediction at each node is the mean of the response variable. This process is repeated, sequentially selecting covariates and split points, until each terminal node contains less than a specified number of observations. Since individual trees are sensitive to overfitting and generally poor at prediction, random forest algorithms average predictions across regression trees. Individual regression trees within random forest models are fit to a bootstrap sample of the data and explanatory covariates. Bootstrap resampling and averaging the predictions from many trees reduces variance without increasing bias, which allows for a large number of highly nonlinear effects to be incorporated while minimizing overfitting. This characteristic is particularly important in datasets such as ours with spatial and temporal structure, which will result in significant autocorrelation unless those processes are incorporated. Machine learning models are tuned by comparing the performance of different suites of hyperparameters, which control an algorithm’s learning process, using cross-validation. Here, we blocked datasets by individual during cross-validation so that detections from the same tag would not inform both training and testing datasets during model development [56].

We compared machine learning algorithms and alternative hyperparameters using 8-fold cross-validation. Model selection and hyperparameter tuning excluded tags deployed in 2022 and randomly assigned data to training or testing groups based on individual blocks. For each model, we evaluated predicted performance using three alternative transformations for observed depth data: untransformed (bounded by zero and bottom depth within a detection radius), a bathymetric depth ratio (observed depth divided by the maximum depth within a receiver’s detection radius; bounded by zero and one), and a logit transform of the bathymetric depth ratio (approximately normally distributed). We compared alternative model structures using root mean square error (predictions based on transformed response variables were back-transformed). We also tested predictions from our 2019-2021 data model fit to the 2022 deployment data as an independent test of future predictive performance. Additional details in Online Supplement.

We fit machine learning models using the caret [57] and ranger [58] R packages. We interpolated a small number of missing ROMS and lipid content estimates (details in Online Supplement) and transformed categorical variables (day-night and maturity stage) to dummy variables prior to model fitting. We quantified the relative explanatory power of different variables by calculating the decline in model performance (percent mean squared error) when a given covariate was excluded from the model (i.e., the performance of trees that included a given variable relative to those that did not).

Random forest models and other machine learning algorithms are most commonly used for prediction. Since covariate effect sizes cannot be evaluated using parameter estimates, we used random forest models for ecological inference by generating conditional predictions to evaluate how Chinook salmon depth varies as a function of one, or multiple, explanatory variables. We emphasize that conditional predictions are not intended to forecast individual depth, but rather to represent a range of plausible vertical distributions, under specific conditions, based on the observed data and fitted model. Our analytical framework provides a suitable means of identifying which covariates are the strongest predictors of vertical habitat use and evaluating how multiple covariates interact with one another. However, alternative techniques, such as generalized additive models or generalized linear models may also be used to estimate effect sizes for covariates (additional details below).

Ultimately, we generated a) non-spatial conditional predictions that focused on the effect of single covariates with all other covariates (including spatial location) held at reference values (median for continuous and 0.5 for dummy variables) and b) conditional spatial predictions that used a grid of cells (1 $\times$ 1 km resolution) with relevant spatial attributes (bottom depth, slope, distance to shore) and all other non-focal variables fixed at reference values (median values except for year day fixed to July 30, dynamic ocean variables fixed to estimates for July 30, 2020, and thermocline depth fixed at its mean value for July). Since machine learning models capture non-linear relationships among large numbers of covariates, we also generated conditional spatial contrasts by calculating the difference in spatially explicit predictions, which allowed us to visualize spatially varying effects.

To quantify uncertainty, we generated infinitesimal jackknife confidence intervals [59] and among-tree quantile prediction intervals using the ranger package [58]. All analyses were completed in R 4.2.1 [60].

Although machine learning models are flexible, they are not a replacement for a sufficient number of independent and representative samples. While our model accounted for temporal autocorrelation at seasonal scales, it did not address subdaily autocorrelation explicitly. Additionally, our data were unbalanced, with a relatively large number of detections associated with a small number of tags (Additional file 3: Fig. S7). Although bootstrap resampling and individual blocking during cross-validation will mitigate these issues to some degree, we completed a series of sensitivity analyses to evaluate their effect on our conclusions (Additional File 1). We fit two hierarchical generalized additive models and three random forest regression models that differed in model form, data preprocessing, and data weighting. We evaluated the relative performance of each model based on residual temporal and spatial autocorrelation, as well as out-of-sample predictive performance and bias. We also compared conditional predictions between models to evaluate qualitative differences on inference. See additional details in the Online Supplement.

## Results

Immature fish (i.e., those observed in the marine environment after November 15 or with a median posterior probability of being classified as mature less than 0.5) were smaller and had a lower lipid content ($66.1\pm 5.54$ cm and $7.24\pm 1.13$ % wet weight; n = 41) than mature fish ($78.6\pm 8.73$ cm and $8.12\pm 1.67$ % wet weight; n = 420). Fork length, lipid content, and the relative proportion of immature fish varied among stocks (Additional file 3: Fig. S2).

We used 43,627 detections with valid depth data from 338 Chinook salmon tagged between 2019 and 2021 to train and test the depth model using cross validation. We included data from 319 mature (median maturation probability greater than 0.5) and 26 immature individuals in the training dataset (sum is greater than 338 because seven individuals provided information as immature and mature individuals). An additional 5,466 detections from 17 mature individuals tagged in 2022 were used to qualitatively evaluate model performance. The number of detections per tag ranged from 1 to 5,987 and tags provided detection data for up to 437 days. There were substantial differences in the number of detections provided by each tag and observed by each receiver (Additional file 3: Figs. S3, S7). Detection depth of Chinook salmon (hereafter depth) ranged from the surface to 352 m, with a median of 28 m and deeper detections among immature fish (Additional file 3: Fig. S4). Chinook salmon were detected in both surface waters and along the sea floor (Additional file 3: Fig. S4). Chinook salmon moved through a wide range of temperatures (6-18 $\hbox {C}^{\circ }$; Additional file 3: Fig. S5) and dissolved oxygen concentrations (<1-12 mg/l, Additional file 3: Fig. S6).

**Fig. 2:**
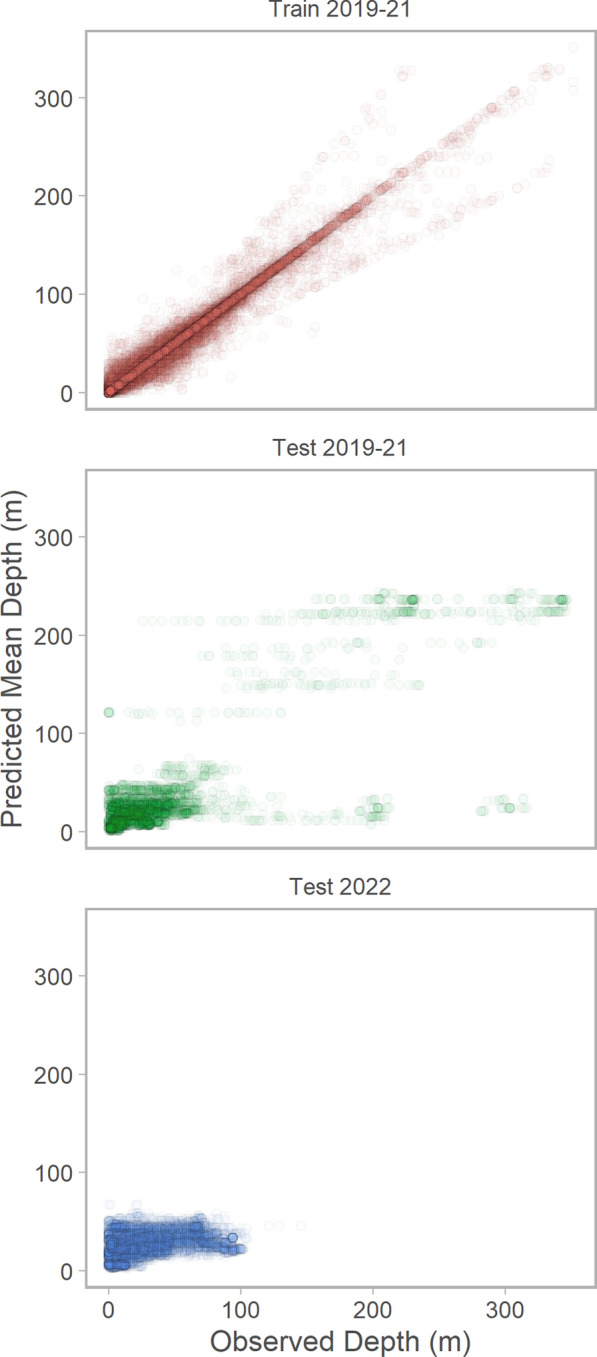
Observed and predicted Chinook salmon depth showing 2019-21 training data from 8-fold cross validation used to fit the initial random forest regression model (red; 80.1% of detections), 2019-21 out-of-sample testing data from 8-fold cross validation (green; 8.8% of detections), and out-of-sample testing data from 2022 tag deployments (blue; 11.1% of detections)

The best-supported machine learning model was a random forest regression model fit to bathymetric depth ratio (i.e., observed depth scaled by the maximum bottom depth within the receiver’s detection radius; Online Supplement). The model included 1000 trees, evaluated 17 variables per tree, and identified split points randomly following a uniform distribution (i.e., extremely randomised trees [61]). The model had unbiased predictive performance with in- and out-of-sample data and high predictive accuracy with in-sample data ($r^2=0.85$); however, root mean square error increased with out-of-sample data, particularly with data associated with detections from 2022 tag deployments (Fig. 2). The proportion of observations within the model’s 95% prediction interval was 99% for the training data, 87% for the 2019-21 testing data, and 91% for the 2022 testing data.

**Fig. 3:**
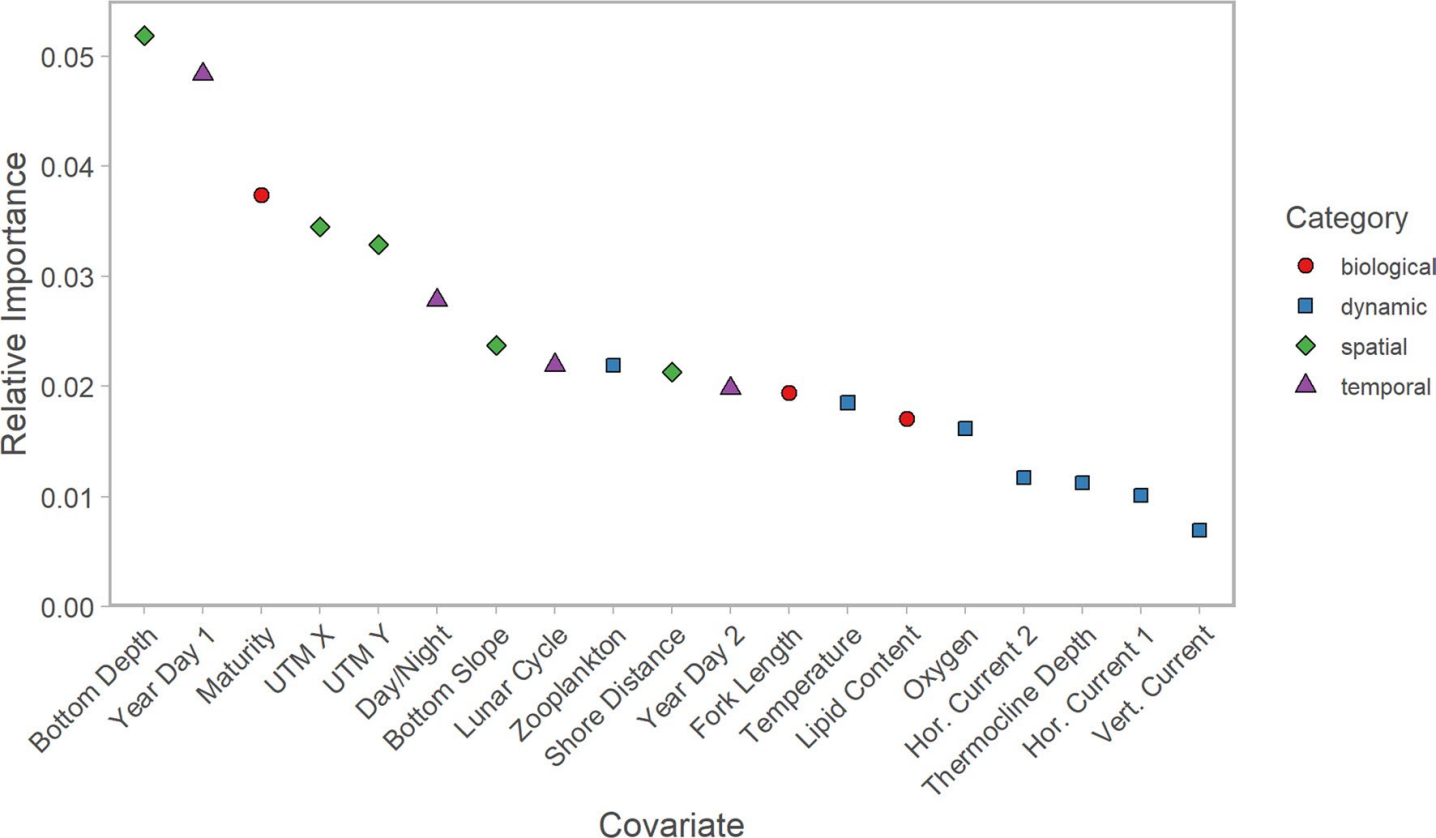
Relative importance of explanatory variables in random forest regression model predicting Chinook salmon mean bathymetric depth ratio. Importance is quantified as the difference (mean among trees) in root mean square error when a given variable is excluded during out-of-bag prediction. An uncorrelated random variable will have a difference of zero. Colors represent categories of explanatory variables

Each variable provided predictive information to the random forest regression model and improved its accuracy; however, the strongest predictors of Chinook salmon depth distribution were bottom depth, one of two composite calendar day variables, maturity stage, and location. The remaining temporal, spatial, and individual variables, as well as a subset of oceanographic variables, had more modest contributions. Current strength and thermocline depth had the poorest predictive performance (Fig. 3). Stock identity covariates also had weak predictive power (Additional file 3: Fig. S8), and the remaining results focus on the model that excluded stock identity effects to improve interpretability.

**Fig. 4:**
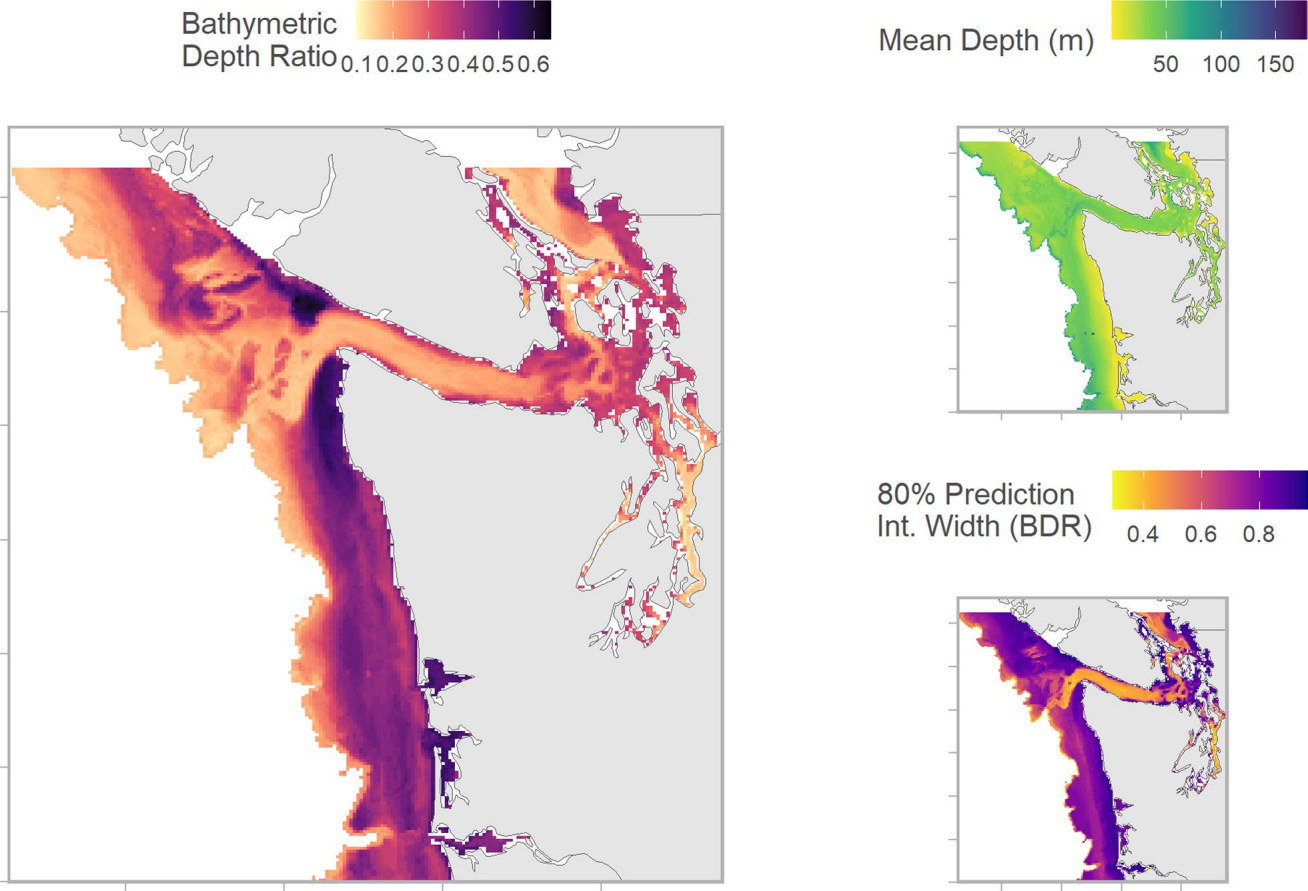
Mean bathymetric depth ratio (left), mean depth (top right), and bathymetric depth ratio 80th percentile prediction interval width (bottom right) of Chinook salmon from random forest regression model. For bathymetric depth ratio, zero represents the surface and one the seafloor. Predictions are conditional effects of spatial processes, with year day fixed to 211 (i.e., July 30) and dynamic oceanographic variables fixed to estimated values for July 30, 2020. All other temporal and biological variables are fixed at median values or 0.5 for dummy variables

Generally Chinook salmon were predicted to occupy the top 20-40% of the water column, relative to bottom bathymetry (Fig. 4). The cumulative influence of bottom depth, spatial location, bottom slope, and distance to shore resulted in strong spatial patterns in vertical distribution. Bathymetric depth ratios were shallowest in western Juan de Fuca Strait, the southern Strait of Georgia, and Puget Sound (Fig. 4). Predicted depth was markedly deeper—50-60% of water column depth—on shelf habitats off the Washington coast and portions of coastal WCVI (Fig. 4). Back-transformed predictions (i.e., depth in meters) were deepest in the southern Strait of Georgia and in the canyons near the western portion of Juan de Fuca Strait (Fig. 4). The prediction intervals associated with the best performing model were relatively wide, consistent with substantial variability among and within individuals (Fig. 4, Additional file 3: S4).

**Fig. 5:**
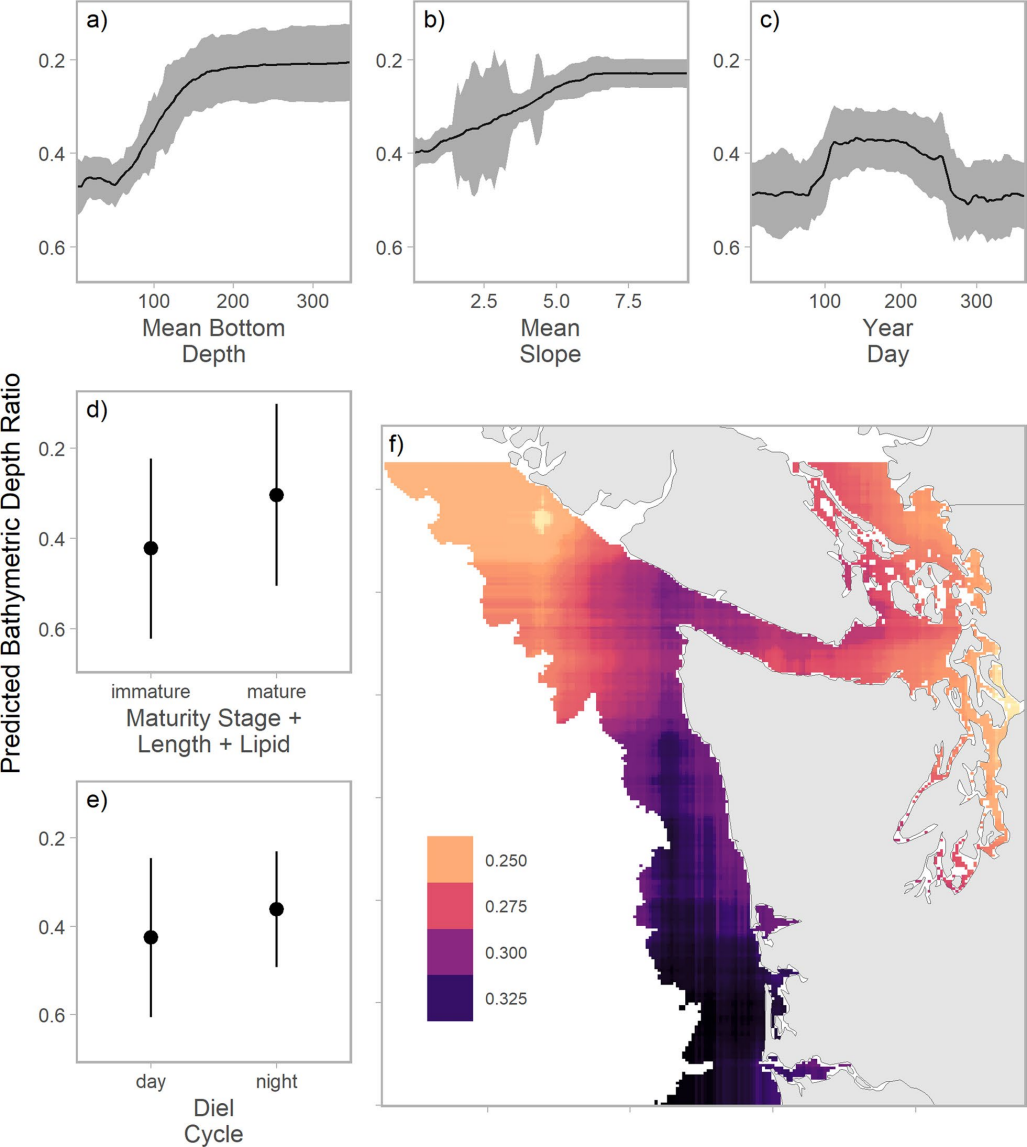
Conditional effects on Chinook salmon bathymetric depth ratio (zero at surface and one at sea floor) of: bottom depth **a**, bottom slope **b**, day of detection **c**, maturity stage/fork length/lipid content **d**, day/night **e**, and easting-northing **f**. Conditional effects represent median random forest regression model predictions (lines or point), assuming all other variables are fixed to reference values (i.e., medians for continuous covariates and 0.5 for dummy variables). Predictions based on maturity stage assume stage-specific mean values for fork length and lipid content. Predictions based on location exclude the effect of spatially correlated variables (i.e., mean bottom depth, mean bottom slope, distance to shore, dynamic oceanographic features). Ribbons and whiskers represent 95% jackknife confidence intervals

Many of the covariates we considered had nonlinear effects on depth. Chinook salmon were more likely to occupy depths closer to the bottom in shallower waters and when bottom topography was relatively flat (Fig. 5, Additional file 3: S4). We found evidence of cyclical, seasonal changes in Chinook salmon depth, where fish were deeper during winter months (Fig. 5). Seasonal changes in mean depth were rapid, occurring over one to two weeks during April (when fish moved to shallower waters) and September (when they returned to deeper waters). Immature fish tended to have deeper distributions than mature fish, particularly after accounting for correlations between maturation stage, size, and lipid content, and nocturnal predictions were shallower than diurnal (Fig. 5). Finally, after accounting for the effect of other spatial covariates (e.g., bottom depth, slope, distance to shore, dynamic oceanographic variables), bathymetric depth ratio deepened along the coast of Washington and in Juan de Fuca Strait (Fig. 5).

**Fig. 6:**
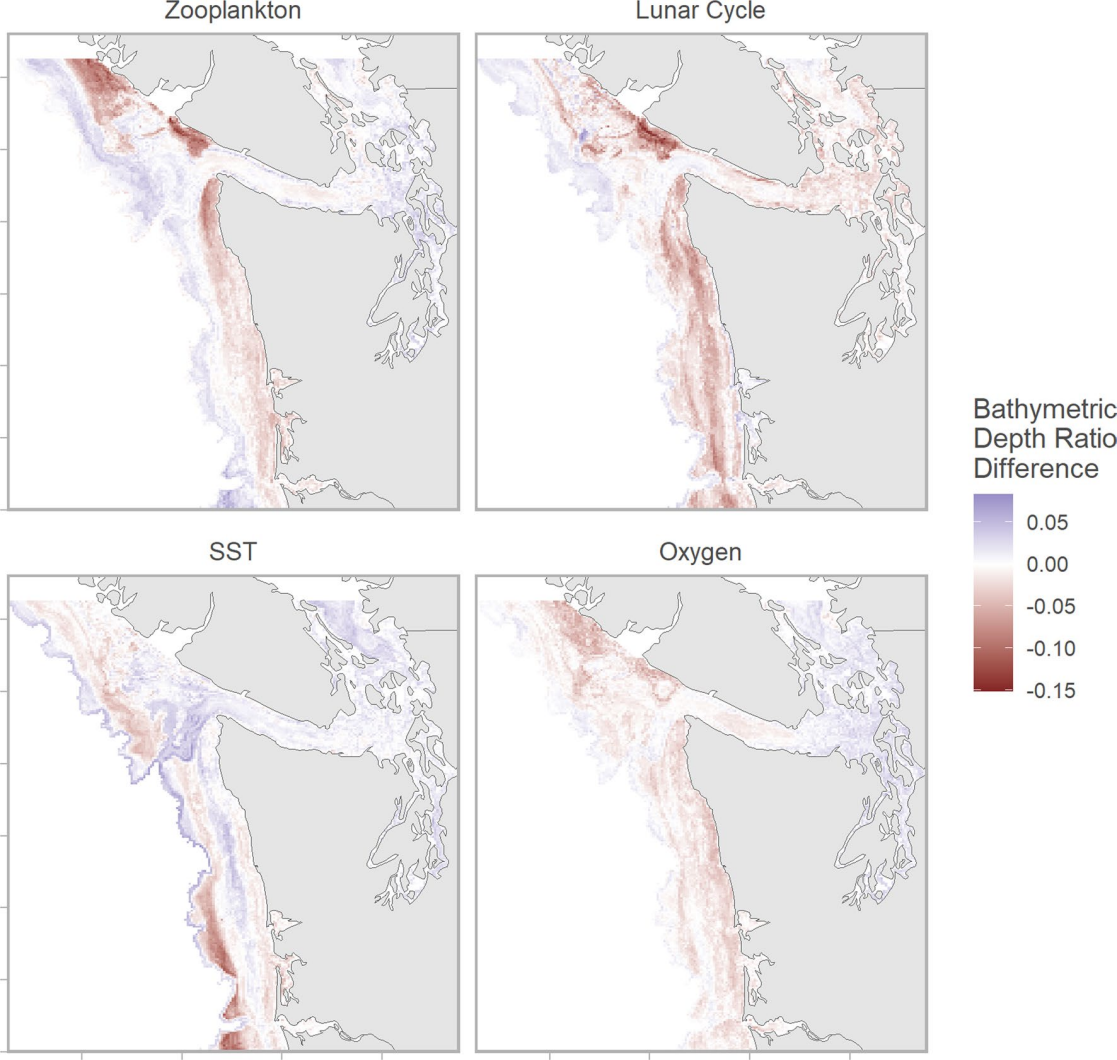
Random forest regression model predicted conditional differences in bathymetric depth ratio across the study area. Maps show predicted differences when: sea surface zooplankton concentrations are 1 SD below or above their summer average (top left); the moon is 0% and 100% illuminated (top right); sea surface temperatures are 1 SD below or above their summer average (bottom left); and sea surface oxygen concentrations are 1 SD below or above their summer average (bottom right). Relative differences were calculated as the difference between predictions (e.g., 0% and 100% illumination) in a given spatial cell. Negative (red) values represent greater predicted depth with greater lunar illumination, temperature, zooplankton concentration, or oxygen concentration. Non-focal spatial covariates were fixed at July 30, 2020 values and the remaining covariates were fixed at median values

The effect of the remaining covariates was generally modest—less than 5% over the range of the observed covariate. However, considering covariates in isolation can obscure interactions between spatial processes and other variables that result in stronger localized effects. For example, the distributions of Chinook salmon deepened when zooplankton concentration, lunar illumination, and oxygen concentration increased, while the effects of sea surface temperature were spatially variable (Fig. 6). Environmental effects were generally constrained to portions of the continental shelf.

## Discussion

Variation in individual depths provides information on the interaction between multiple ecological processes that regulate habitat use in marine species. We used observations from more than 300 adult Chinook salmon tagged during a four-year period to disentangle the effect of spatial, temporal, physical, and biological processes on vertical distributions. Tagged individuals ranged from the surface to nearly 400 m in depth, but were typically distributed near the middle of the water column approximately 25 m below the surface. Chinook salmon encountered a wide range of thermal habitats, from six to $18~\hbox {C}^{\circ }$, and intermittently hypoxic conditions. Within the top performing model a subset of covariates representing bottom depth, spatial, seasonal, and maturation stage effects were most strongly correlated with Chinook salmon depth distributions. While the model performed relatively well even with held-out training data, its prediction intervals were wide emphasizing substantial variability among and within individuals in vertical habitat use.

### Ecological drivers of depth distribution

Our results are consistent with Chinook salmon depth distributions reflecting prey availability and associated foraging behaviours. A position within the middle of the water column is consistent with a vertical ambush strategy whereby larger-bodied piscivores attack pelagic prey from below [62]. Moreover, several of the top-ranked covariates have clear linkages to prey availability. Complex bottom bathymetries increase bio-physical coupling as nutrients are deflected from deeper waters, resulting in greater primary productivity and concentrating zooplankton and forage fish [16, 63]. In this study, bottom depth and slope were correlated with individual depth, and also with one another, suggesting these variables are proxies for bottom complexity. Furthermore, steep dropoffs, reefs, or pinnacles on the west coast of Vancouver Island often have greater Chinook salmon abundance and are commonly targeted by fishers (C. Freshwater and B. Hendriks, unpublished data). In this paradigm, residual spatial variation likely represents foraging hotspots associated with variables excluded from the model (e.g., bottom substrate, locations of tidal outflow).

Seasonal changes in Chinook salmon depths also mirror changes in prey species behaviour. Forage fish in the northeast Pacific, particularly Pacific herring *Clupea pallasii* and northern anchovy *Engraulis mordax*, become increasingly bottom-oriented during the winter [64–66] and are key prey of Chinook salmon [39, 40]. We also identified localized correlations between zooplankton concentration and depth, which provides additional evidence that Chinook salmon distributions covary with productivity in rearing habitats.

Although we believe changes in vertical distribution are most consistent with foraging behaviour, Chinook salmon may also moderate their depth in response to predation risk. Resident killer whales prey upon Chinook salmon throughout the year [67, 68] and have similar depth distributions to Chinook salmon [13]. Chinook salmon were deeper relative to bottom bathymetry near western Juan de Fuca Strait and the southern Strait of Georgia, which have been identified as resident killer whale foraging hotspots [69]. Both cetacean and pinniped habitat use is also correlated with bottom depth and topography [70, 71], presumably because these features concentrate prey. Moreover, the depth of salmon sharks *Lamna ditropis*, another salmon predator, changes seasonally [72], suggesting changes in depth during winter may have knock-on effects across trophic levels.

The distributions of mature Pacific salmon shallow during marine migrations as they use olfactory cues to home to natal streams [27, 28]. Immature Chinook salmon, which were also smaller and had a lower lipid content than mature individuals in this study, had deeper distributions, even after accounting for seasonal effects. The spatially varying effects of zooplankton concentration we observed were also consistent with fish responding more strongly to productivity in rearing areas, such as La Perouse Bank and coastal Washington, than in migratory locations such as the southern Strait of Georgia. Thus, our results are consistent with mature individuals becoming increasingly surface oriented as they migrate towards terminal areas. Notably, stock identity was a poor predictor of variation in depth distribution, presumably because other model covariates accounted for stock-specific traits such as body size and migration route.

We did not find strong evidence that oceanographic conditions regulated Chinook salmon distributions. Zooplankton concentration, sea surface temperature, and oxygen concentration had modest performance, but their predictive power was substantially weaker than other covariates, and the effects of thermocline depth and current velocity were weaker still. Consistent with previous Pacific salmon tagging studies, we found tagged individuals encountered a wide range of environmental conditions [29, 38]. Chinook salmon may adopt strategies similar to other pelagic piscivores, whereby physiologically taxing habitats are used to concentrate prey for capture [62, 73].

### Divergent patterns among marine ecosystems

While we found spatial and seasonal variables were the strongest drivers of variation in depth distribution, previous work on Pacific salmon and other large pelagic fishes has emphasized the importance of processes occurring at shorter temporal scales. We believe that our divergent results are due to the ontogeny of tagged fish and their habitats. Previous work on Pacific salmon has demonstrated that vertical and horizontal habitat use is moderated by temperature and oxygen. Many of these studies have focused on individuals that have ceased feeding to undergo terminal migrations. Individuals in terminal areas use surface waters for orientation [27, 28] and deeper habitats as thermal refugia [29, 74, 75]. Conversely, the Chinook salmon we tagged did not enter freshwater for weeks to months (or over a year in the case of immature fish). As a result, they were continuing to forage and may have been less likely to exhibit strong responses to physical gradients [36, 38].

While there is evidence of temperature or oxygen constraining Chinook salmon habitat use in California Current habitats [32, 76], temperatures in this region are considerably warmer than those off southern Vancouver Island and northern Washington and oxygen may be more limiting due to stronger seasonal upwelling [4, 77]. Indeed, Sabal et al. 2023 reported that a) the warmest temperatures they reported were not observed in the northern portion of their domain (which overlapped with our study area) and b) the Chinook salmon populations they observed in northern regions did not show strong responses to temperature [32]. We found that Chinook salmon showed moderate responses to sea surface temperature and oxygen concentration in shelf habitats and were consistently deeper in the water column on the Washington continental shelf than elsewhere. Both patterns suggest that the effects of dynamic oceanographic conditions vary spatially.

More generally, our tagging data can be used to compare the vertical movements of Chinook salmon to well-studied species such as billfishes, tunas, and sharks (hereafter large pelagics). Chinook salmon and large pelagics are in many ways ecologically similar. They are predominantly piscivorous, undergo extensive migrations, and often exhibit diving behaviour. Large pelagics such as dolphinfish *Coryphaena hippurus* and silky sharks *Carcharhinus falciformis* are largely constrained to the epipelagic. Blue sharks *Prionace glauca* and bigeye tuna *Thunnus obesus*, however, dive into the mesopelagic where they encounter cooler temperatures and hypoxic conditions [7]. Similar to the latter species, we found Chinook salmon moved through a wide range of temperatures, intermittently co-occurred with very low oxygen concentrations, and were not constrained by the thermocline. Like some large pelagics [7], Chinook salmon were shallower at night and distributed deeper in the water column when light levels increased, presumably due to improved foraging efficiency at depth. Although the effect of diel period was weaker than many other covariates, the forage fish that Chinook salmon prey upon do migrate to shallower depths at night [78], which may elicit a behavioural response particularly before Chinook salmon begin their terminal migrations.

Unlike large pelagics, however, Chinook salmon were not strongly associated with thermocline depth or horizontal and vertical currents, a proxy for mesoscale features [26]. We suggest these differences are associated with the marine habitats each group occupies, as well as their associated physiological adaptations. Large pelagics are warm water species that may dive to colder habitats to forage, but return to the surface to thermoregulate [7]. As a result, large pelagic tagging studies typically occur in offshore regions with substantial stratification [7, 10], a more extreme version of the California Current Chinook salmon studies described above. Conversely, Chinook salmon are cold water tolerant species with an extensive marine distribution [39, 79]. Our study area, which occupies the central portion of the species range, includes regions that are relatively well-mixed due to estuarine circulation (Juan de Fuca Strait) or seasonal upwelling (coastal Washington and WCVI) and generally shows less vertical structure in temperature than tropical regions [4].

### Limitations and conclusions

Our analysis of Chinook salmon depth distributions using acoustic telemetry data has several limitations. First, we did not estimate horizontal and vertical habitat use simultaneously. A shift in Chinook salmon distributions from migratory corridors to productive habitats, coincident with increases in depth, would be strong evidence that foraging decisions moderate seasonal variation; however, developing fully three-dimensional models was outside the scope of this work.

Second, our estimates of vertical habitat use include several sources of uncertainty. Since fish locations could not be identified within the detection radius of a receiver, our model integrated variation within that radius. Similarly, the tag pressure sensors had an estimated precision of several meters, resulting in substantial observation error. Observation error is unlikely to bias our results, however large detection radii and imprecise estimates of depth will inflate residual error within our model predictions. If observation error could be reduced or accounted for statistically, then we may be better able to resolve fine-scale behaviours. While ROMS model outputs accurately capture relatively large-scale variability in oceanographic conditions, they likely do not fully reflect granular environmental processes that serve as cues for individual fish. Size and lipid content at the time of tagging are reasonable short-term proxies for body condition, but our model did not account for individual growth. Size, lipid content, and other traits also differ among Chinook salmon stocks. While including stock identity did not improve model performance, it remains unclear to what extent differences among Chinook salmon in vertical distribution are due to individual variation as opposed to stock-specific behaviours that covary with traits such as size or migration route.

Third, we tagged a relatively small number of individuals, with the number of observations varying among individuals. Despite unbalanced data, our sensitivity analyses suggested that our analytical approach had better performance than comparable generalized additive models that accounted for among individual variation hierarchically via random intercepts. Additionally, random forest models that accounted for unbalanced sampling via data weighting provided similar predictions, but with greater certainty. Thus the uncertainty associated with among individual variability appears to be adequately captured by our model. Perhaps more importantly, the populations we tagged, and their associated habitats, represent only a fraction of the ecological diversity within Chinook salmon. Chinook salmon behaviour may vary regionally or among life-history types that were not well sampled here (e.g., populations with yearling life histories that mature offshore). More generally, we could only sample depth distributions in locations and times where receivers were deployed. The accuracy of our model’s predictions will be reduced if habitat use differs in unmonitored locations or times. Indeed, model performance worsened when data from 2022 tag deployments were compared to predictions from the model trained on 2019-21 tag deployment data. We note, however, that challenges associated with generalizing from ecological studies are widespread and that our sample size, in terms of individuals, is larger than many comparable tagging studies (e.g., median among studies cited here was 19).

Unlike open ocean pelagic environments, we found that vertical habitat use on the continental shelf was moderated at relatively coarse scales by interactions between static spatial variables and seasonality. Processes occurring over shorter temporal periods had weaker or more localized effects. Variation in Chinook salmon depth distributions provides both challenges and opportunities for management. On the one hand, strong seasonal effects coupled with a broad vertical distribution suggest catchability varies, complicating the interpretation of fisheries-dependent data [12]. On the other, evidence that depth varies spatially, seasonally, and ontogenetically could guide interventions intended to constrain directed and incidental harvest. Previous work has highlighted how trade-offs between growth, predation risk, and reproductive success can influence habitat selection in aquatic species [24, 80]. Our analysis provides a framework for extending this work into difficult-to-observe pelagic ecosystems by linking tagging data, dynamic oceanographic models, and spatially explicit models.

### Supplementary Information

**Additional file 1.** Supplementary analyses evaluating alternative model structures.

**Additional file 2.** Conservation units and stock aggregates that were tagged.

**Additional file 3.** Supplementary figures.

## Data Availability

The data and code underpinning these analyses are available from https://github.com/pacific-salmon-assess/chin_depth and have been archived with Zenodo at https://zenodo.org/doi/10.5281/zenodo.10698892.
