# Supplementary material for: Chinook salmon depth distributions on the continental shelf are shaped by interactions between location, season, and individual condition
Source: Mov Ecol. 2024 Mar 15;12:21. doi: 10.1186/s40462-024-00464-y (PMC11337652; doi:10.1186/s40462-024-00464-y)
Supplement: Supplementary file 1 — Additional file 1. Supplementary analyses evaluating alternative model structures. [file 40462_2024_464_MOESM1_ESM.docx]

Supplementary Analyses

In addition to the supplementary analyses to determine the best performing machine learning algorithm, we conducted analyses to evaluate the sensitivity of our results to assumptions about data structure and the structural form of models. We considered five different model forms. First, the random forest regression model described in the main text (rf_main). Second, a random forest regression model that incorporated data weighting via the inverse square root of the sample size (rf_weighted1). Third, a random forest regression model that incorporated data weighting via the inverse of the sample size (rf_weighted2). Fourth, a hierarchical generalized additive model (GAM) that included a subset of covariates (gam_full). Fifth, a hierarchical GAM that was fit to aggregated detections data, but included the full set of covariates (gam_mean). Two types of GAMs were necessary due to slow computation speed given the large number of observations and covariates. Given evidence that models fit to bathymetric depth ratio data outperformed those fit to observed depth data or logit transformed bathymetric depth ratio data (Online Supplement), we fit all models using bathymetric depth ratio as the response.

The weighted random forest models were functionally identical to the random forest model presented in the main text, except we accounted for differences in the number of observations among individuals by weighting data from each tag relative to the total number of detections available for that tag. The goal of sample weighting is to increase the representation of relatively undersampled data, which was achieved here by resampling observations within the random forest regression (i.e. bootstrap samples used to generate individual regression trees), proportionally to each observation’s sample weight (Thum et al. 2017, Cameron et al. 2022). Since the inclusion of sample weights has cascading effects on model fitting, we repeated the hyperparameter tuning procedure for the weighted random forest models and present results from the top model in this section. Sample weighting was performed using the “case.weights” argument within the ranger function of the ranger package (Wright and Ziegler 2017).

Since bathymetric depth ratio data were bounded between zero and one, we fit hierarchical GAMs using a beta distribution and logit link function with parameters μ and ϕ . GAMs model relationships between response and explanatory variables as smooth non-parametric functions (Wood 2017). The first model was fit to the same data as the unweighted random forest regression model and took the form

$$d \sim Beta(\mu,\phi)$$

$$logit(\mu)=f\left( l_{x}, l_{y} \right)+f\left( b \right)+ f\left( t \right)+f\left( s \right)+ \alpha_{p}+\alpha_{i}$$

Where *d* represents mean depth, *f* are basis functions for location *l* with easting *x* and northing *y*, mean bathymetric bottom depth, year day *t*, and maturation stage probability (bounded by 0 and 1) *s*. All basis functions were estimated using smooths characterized by thin plates splines, except for location, which was estimated using a tensor product to estimate the interaction between northing and southing, and year day, which was estimated using a cyclic smoother that ensured estimates for year day 1 and year day 365 were similar (analogous to the approach we used in the main text). We also estimated a fixed intercept for diel period $\alpha_{p}$ and a random intercept for each individual $\alpha_{i}$ to account for repeated observations among individuals. Variance in depth was estimated as

$$\mathrm{var}[d]= \frac{\mu(1-\mu)}{1+ \phi}$$

We note that this GAM contains a subset of the explanatory variables identified as having the greatest effect size in the random forest analysis presented in the main text. We did not include the full suite of variables here due to slow computation speed when fitting the model to the full training dataset (n = 39,441). However, we also fit a second hierarchical GAM that was identical to the first except it estimated additional parametric linear effects for bathymetric slope, fork length, lipid content, distance to shore, lunar illumination, and the full suite of ROMS variables highlighted in the main text. Such a model was tractable because we fit the model to binned detections data. Specifically, we calculated the mean bathymetric depth ratio for each tag at each location within a given hour (n = 8,348). While interactions among variables (e.g., spatially varying effects) arise naturally in random forest models because predictions are averaged across many individual regression trees, we did not estimate similar effects within the GAMs due to computational constraints. We fit both GAMs in the mgcv package (Wood 2011). We note that we also attempted to fit GAMs that accounted for temporal autocorrelation at the subdaily level via an autoregressive residual error term; however, these models failed to converge.

Each model accounted for unbalanced sampling among individuals in slightly different ways. The effective sample size of the rf_main was lower than the total number of detections because the model was constrained by covariates that varied among individuals (i.e. lipid content, fork length, maturation stage) and hyperparameter tuning used blocks among individuals to reduce overfitting. rf_weighted and rf_weighted2 were constrained by these effects as well as the data weighting, which increased the relative importance of observations from more rarely observed individuals. The GAMs included an individual-level effect via a random intercept, as well as additional individual-level covariates (though in gam_full this was only maturation stage), similar resulting in similar among variable constraints to the random forest model.

We evaluated differences among models across four metrics: residual temporal autocorrelation, residual spatial autocorrelation, out-of-sample predictive performance, and conditional predictions across covariates common to all models. As presented in the main text, out-of-sample performance was evaluated using 2022 tag releases (i.e., data from the same tags and years were not used in both datasets). Ideally model performance would be assessed using multiple replicates, adjusting the training and testing data accordingly. However we felt our approach was justified given a truly independent testing dataset was available, and that models took a considerable time to converge.

*Temporal Autocorrelation*

We evaluated temporal autocorrelation by calculating Pearson residuals using the predictions (in real space) from each model, converting the observed data into evenly spaced time series (minute for rf_main, rf_weighted, rf_weighted2 and gam_full; hour for gam_mean), and computing the autocorrelation function for each individual. We then calculated the mean and median autocorrelation coefficient at lag-1 (i.e. AR-1) estimate among individuals for each model.

Subdaily residual temporal autocorrelation was highly variable among individuals, but was generally highest for gam_full (among individuals mean = 0.538, sd = 0.339; Figure S2.1). The GAM fit to hourly binned data was markedly lower (mean = 0.100, sd = 0.380). The standard random forest model had the lowest median temporal autocorrelation estimate (mean = -0.017, sd = 0.257), while the weighted random forest models were slightly higher, but still lower than either GAM (mean = 0.087, sd = 0.304; mean = 0.032, sd = 0.307).


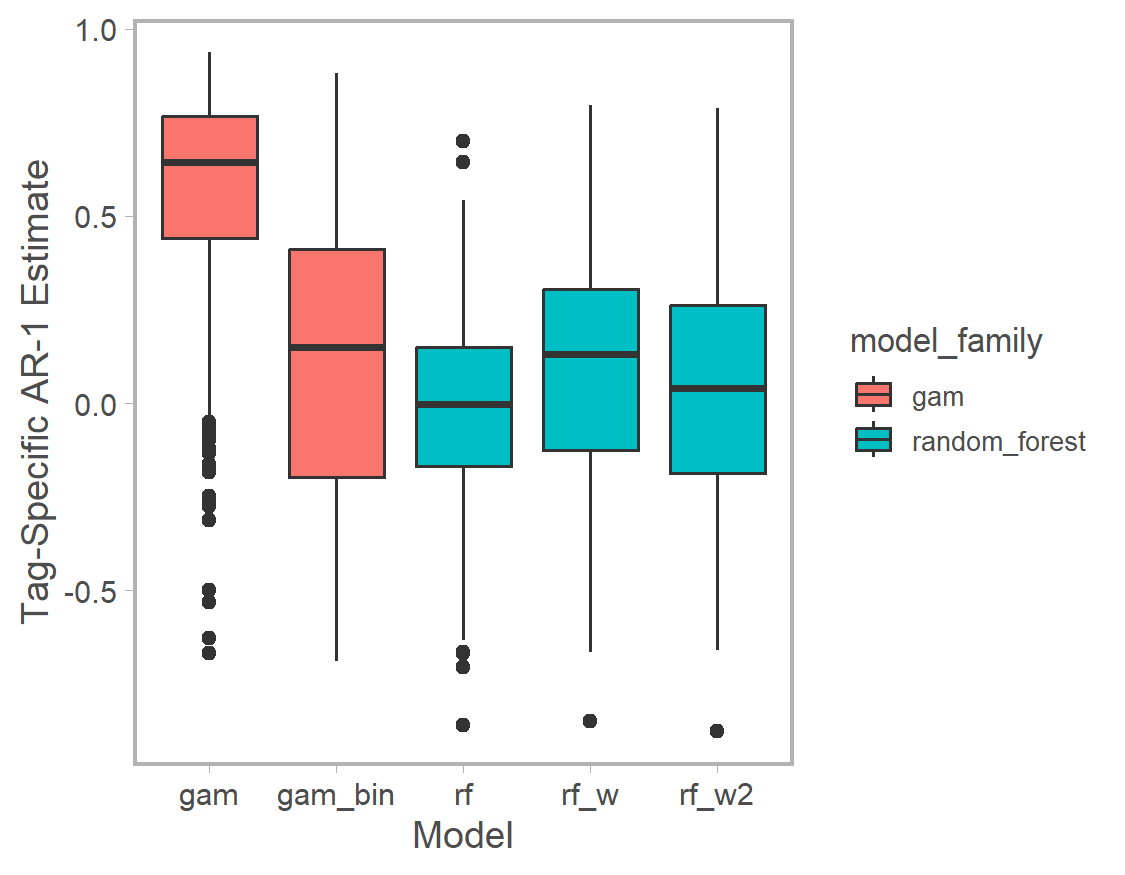


Figure S2.1. Estimate of lag-1 temporal autocorrelation from residuals of fitted models. Time intervals are one minute for all models except gam_bin where the interval is one hour. Boxplots represent the distributions among tags.

*Spatial Autocorrelation*

We evaluated spatial autocorrelation by calculating Moran’s I test statistic for each model’s Pearson residuals (Moran 1950). All estimated coefficients were small (i.e., approached zero); however, the residuals from the gam_full model were statistically significant.

Table S2.1 Moran’s I test statistic of spatial autocorrelation applied to Pearson residuals from five candidate models.

| Model | Estimated Moran’s I Coefficient (SD) | P-Value |
| --- | --- | --- |
| gam_full | **0.033 (0.002)** | **<0.001** |
| gam_bin | 0.004 (0.006) | 0.524 |
| rf | <0.001 (0.002) | 0.972 |
| rf_weighted1 | <0.001 (0.002) | 0.790 |
| rf_weighted2 | 0.003 (0.002) | 0.099 |

*Predictive Performance*

Given the structural differences among models, out-of-sample predictive performance was the most viable way to evaluate each model’s performance. We evaluated accuracy (root mean square error) and bias (mean difference between observed and predicted) of the predicted bathymetric depth ratio for each model after fitting to observations from the 2022 tag deployments. gam_full had the best out-of-sample accuracy (Fig S2.4), but was also the most biased (Fig S2.5). The remaining models had similar accuracy and bias.


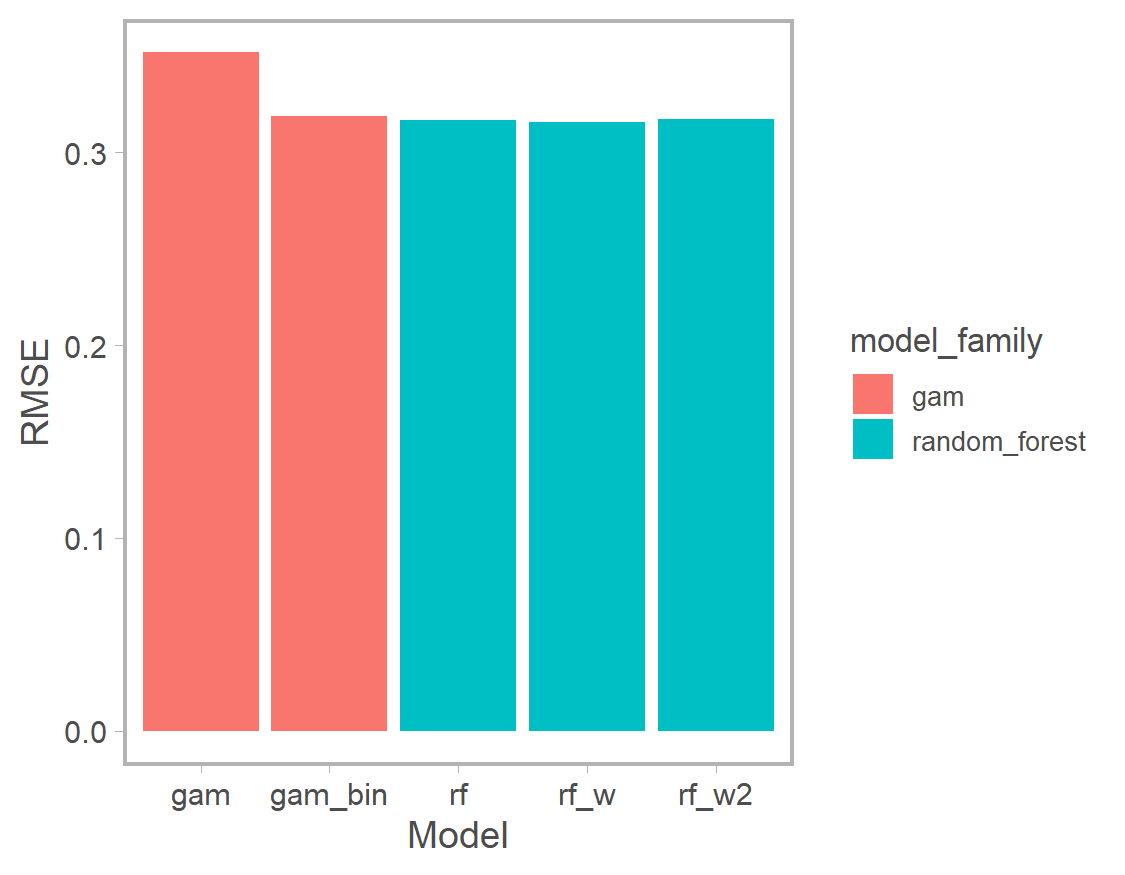


Figure S2.4. Out-of-sample accuracy of each fitted model based on root mean square error. Note that all models were compared to the same hold-out testing data and there was only one replicate, so RMSE is not expected to be centered on zero.


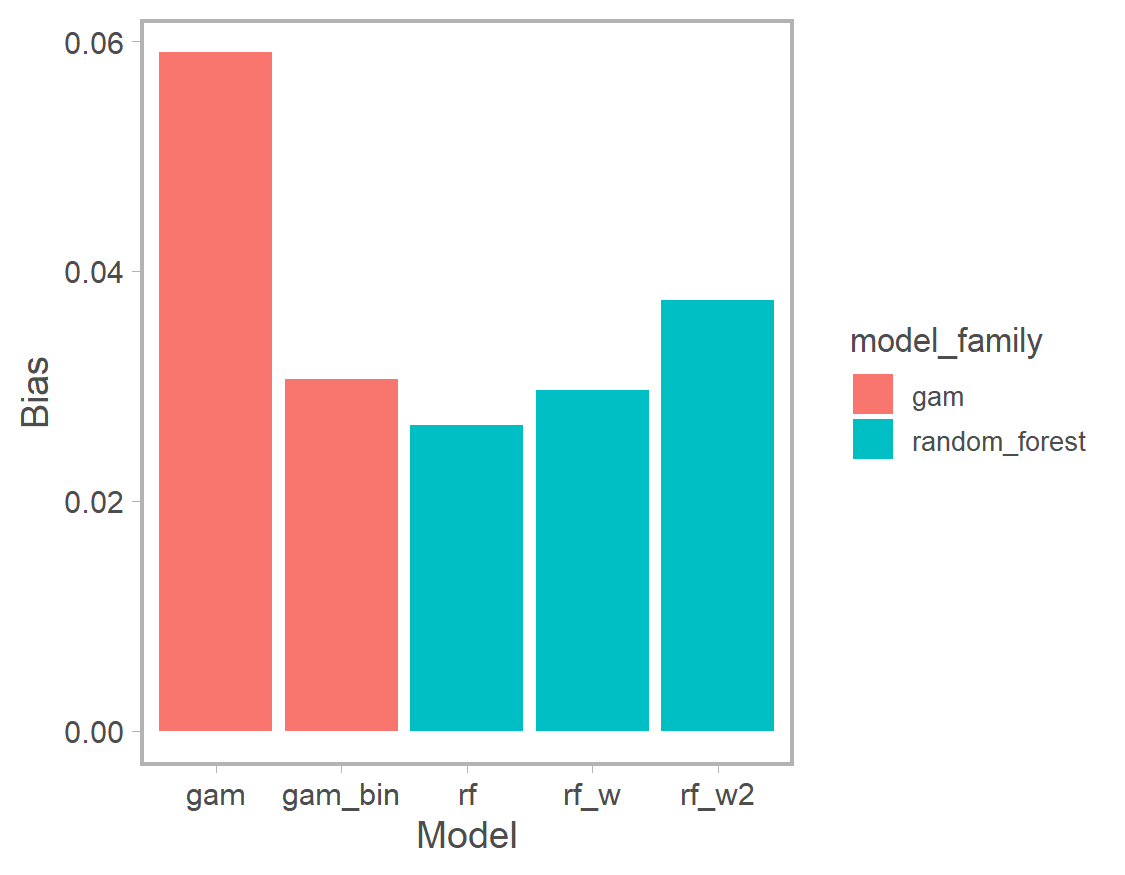


Figure S2.5. Out-of-sample bias of each fitted model based on the average difference between observed and predicted. Note that all models were compared to the same hold-out testing data and there was only one replicate, so bias is not expected to be centered on zero.

*Conditional Predictions*

We drew inference on the relative importance of different covariates based on conditional predictions (i.e. the magnitude of the predicted effect when fixing other covariates at reference values). We evaluated the sensitivity of these inferences to uncertainty in model structure by comparing conditional predictions for the subset of covariates common to all models (and identified as having the greatest predictive power in the primary analysis).

The degree of similarity in model predictions varied among covariates. For example, the directionality and magnitude of bottom depth, year-day, and maturation stage effects were similar among models, although the magnitude and uncertainty of those effects was variable (Fig S2.6, S2.7, S2.8). Conversely, the predicted effect of spatial location differed between random forest models and GAMs due to the complex nonlinear relationship estimated by GAMs (Fig. S2.9, S2.10). The GAM models’ conditional spatial predictions may have been a symptom of model overfitting since they oscillated between fully surface- and fully bottom-oriented, which was not observed in other model fits with any other covariate.

Generally, the GAM fit to aggregated data (gam_bin) and the weighted random forest regression model (rf_w) had the least uncertainty associated with predictions (though this varied among predictor variables). For gam_bin, reduced uncertainty is likely due to data pre-processing, which removed variability at subhourly intervals within a location. For rf_w reduced uncertainty is presumably due to within individual variability being reduced by increasing the sampling rate of less frequently observed individuals. The full GAM also was biased negative relative to the other models for specific covariates (Fig S2.6, S2.8). We note that the random forest model presented in the main text had similarly high levels of uncertainty to the rf_w2 model.


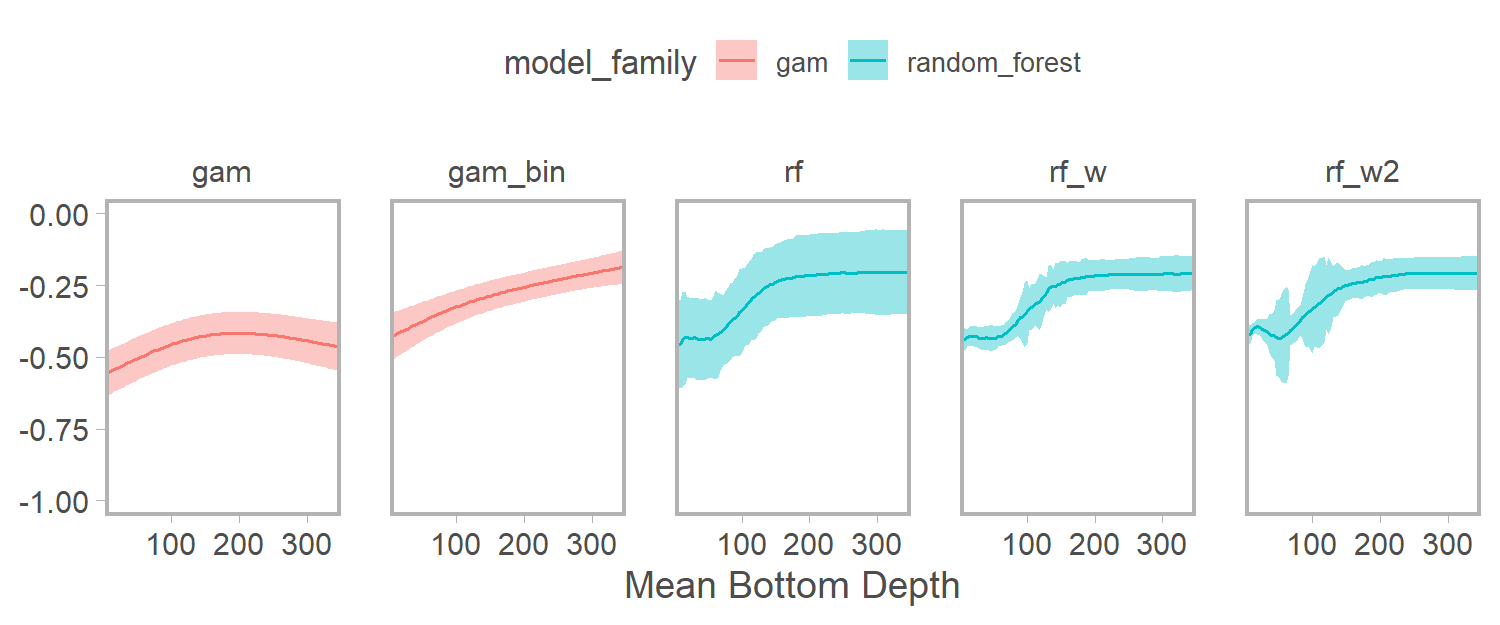


Figure S2.6. Conditional predicted effect of bottom depth on bathymetric depth ratio. Solid lines represent mean (median) prediction and ribbons the 95% confidence interval (quantile interval) for GAMs (random forests).


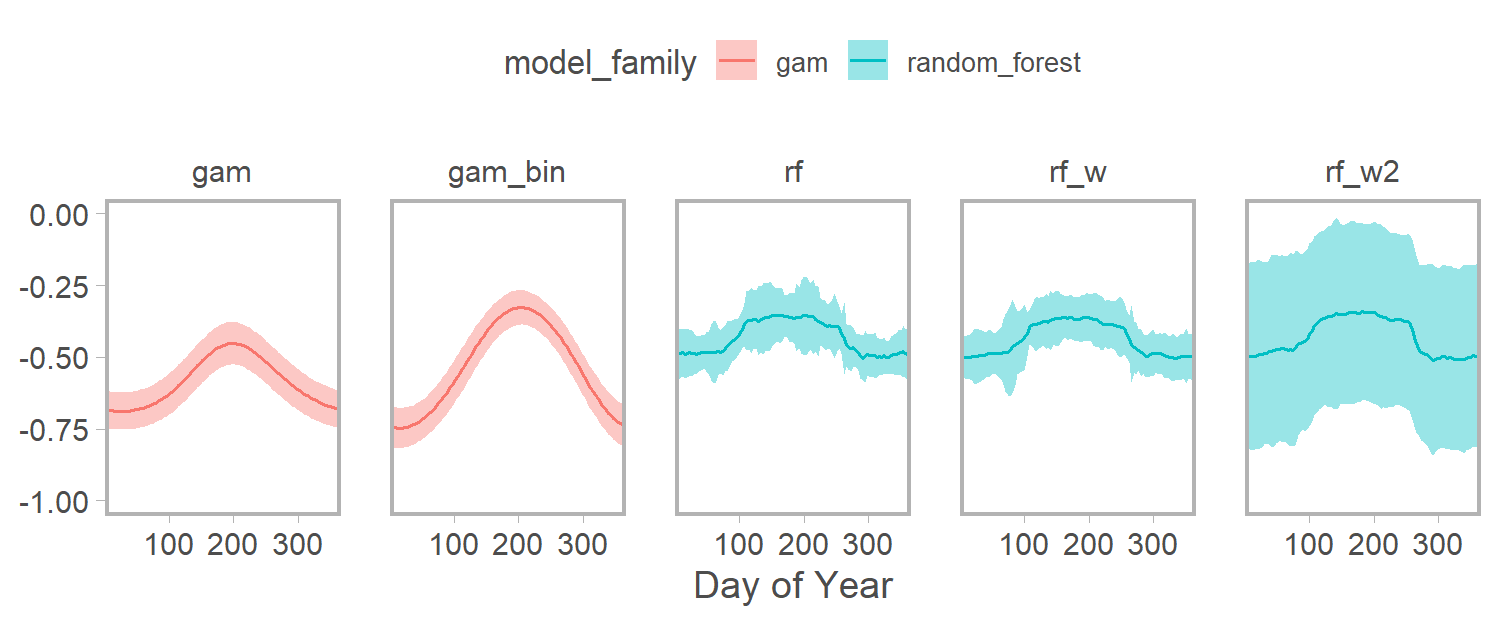


S2.7. Conditional predicted effect of year day on bathymetric depth ratio. Solid lines represent mean (median) prediction and ribbons the 95% confidence interval (quantile interval) for GAMs (random forests).


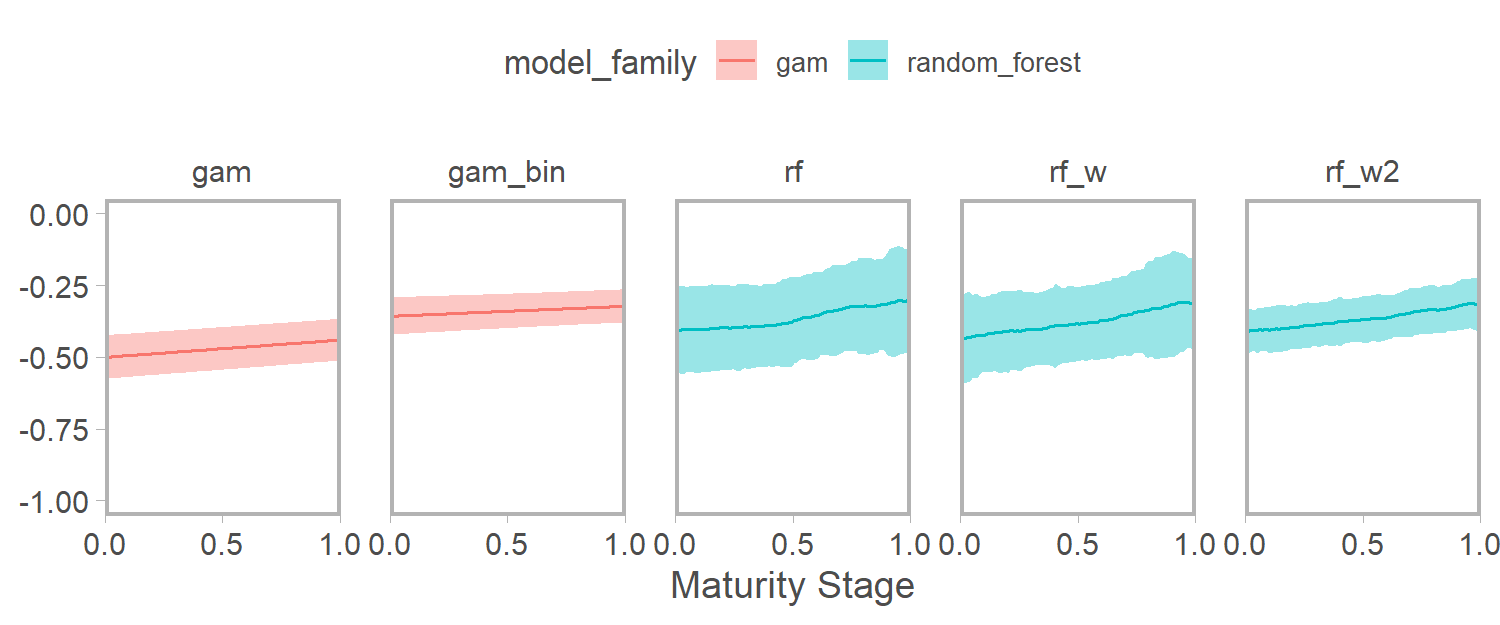


Figure S2.8. Conditional predicted effect of maturation stage on bathymetric depth ratio. Individuals were assigned a probability of zero (one) for known immature (mature) indivdiuals and itnermediate values represent posterior mean prediction for individuals where stage could not be reliably identified with acoustic detections data. Solid lines represent mean (median) prediction and ribbons the 95% confidence interval (quantile interval) for GAMs (random forests).


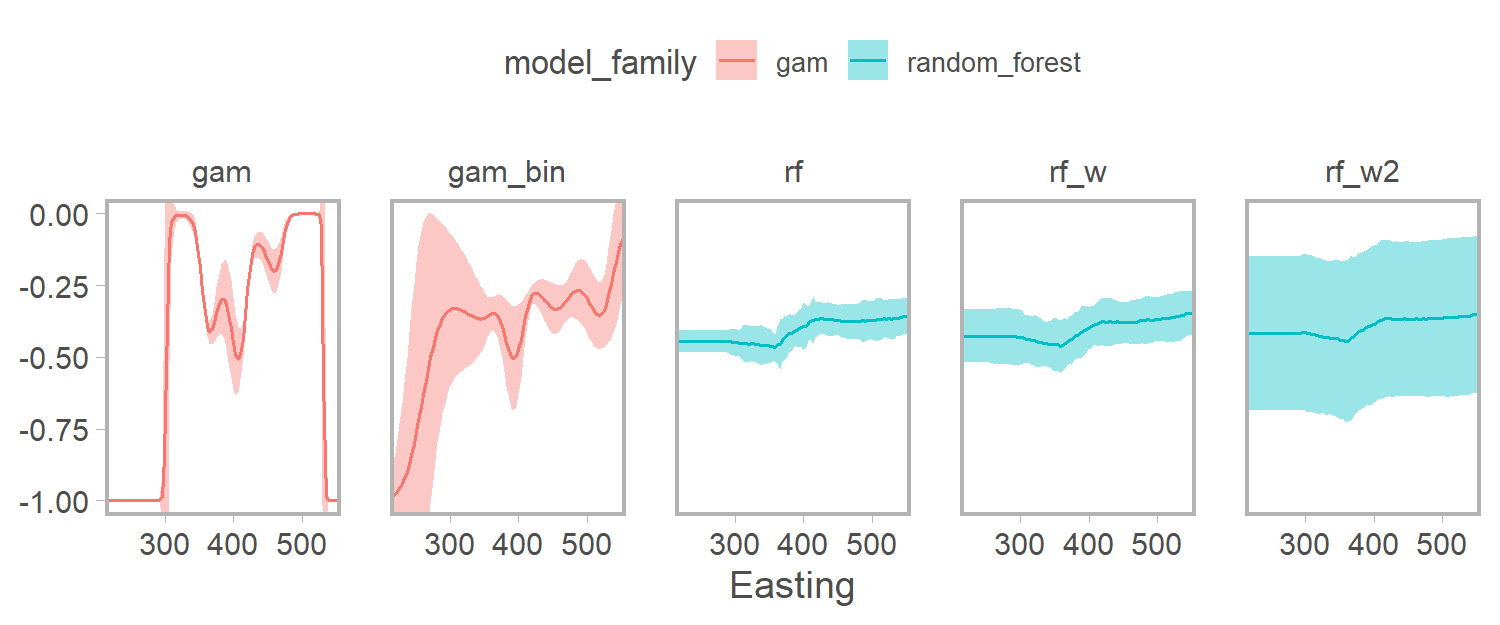


Figure S2.9. Conditional predicted effect of easting on bathymetric depth ratio. Solid lines represent mean (median) prediction and ribbons the 95% confidence interval (quantile interval) for GAMs (random forests).


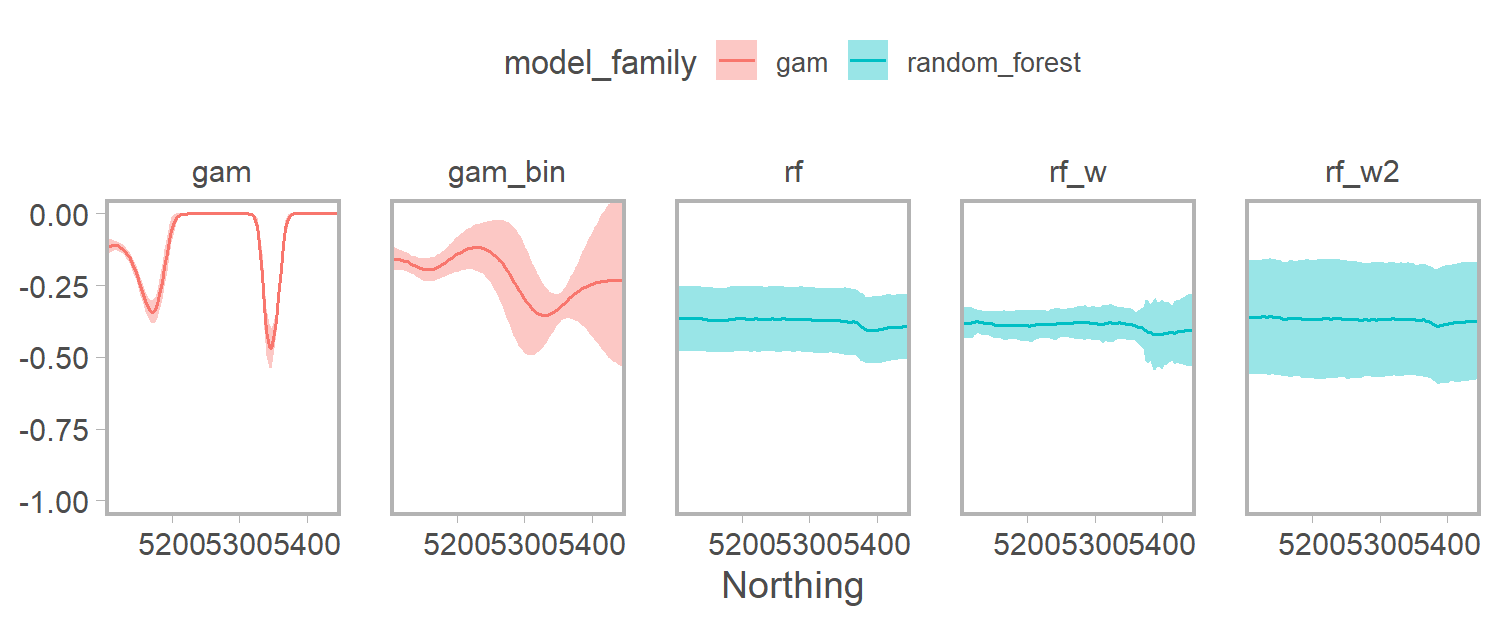


Figure S2.10. Conditional predicted effect of northing on bathymetric depth ratio. Solid lines represent mean (median) prediction and ribbons the 95% confidence interval (quantile interval) for GAMs (random forests).

*Conclusions*

Selecting between traditional models, with explicit assumptions about underlying statistical distributions, and machine learning models, such as random forest, involves trade-offs between structural constraints and model interpretability (James et al. 2017). Evaluating the relative influence of individual covariates in traditional models is relatively straightforward via effect size estimates (though this does become challenging when many covariates are included, non-linear effects are estimated, or non-normal response data are modeled). Conversely, machine learning approaches rely on more opaque metrics (e.g., relative importance) and conditional predictions to evaluate the impact of covariates.

Based on the above sensitivity analysis, the gam_full model is not reliable due to substantial residual spatial and temporal autocorrelation. Additionally, the gam_full model had the greatest out-of-sample predictive bias. While the gam_bin model addresses these shortcomings, it requires substantial preprocessing of the data using relatively arbitrary thresholds (i.e. we used hourly means, but smaller or larger temporal bins may be just as defensible). Aggregating subhourly observations into hourly means results in substantially less variability and, as a result, reduced uncertainty in predictions, which may be unrealistic. Finally, both GAM models displayed evidence of overfitting in spatial effects, which may not realistically represent these latent spatial processes.

The random forest models gave qualitatively similar results to the gam_bin model (except for latent spatial effects) and had similarly low levels of spatial and temporal autocorrelation. Additionally these models did not show extreme oscillations in latent spatial processes and their predictions are more robust to collinearity among covariates, allowing us to consider the full suite of predictor variables without violating assumptions (e.g., high values of concurvity in GAMs associated with multicollinearity). Finally, random forest models automatically account for non-linear additive effects among covariates, which allowed us to evaluate spatially varying effects. It is unlikely that either GAM would converge with these interactions included. Since all three random forest models provided qualitatively similar results, the original model appears to adequately account for unbalanced sampling among individuals. We tend to favor the original random forest model, rather than either of the weighted options, because it does not require arbitrary decisions on how data should be weighted, which can strongly impact outcomes if mis-specified (Cameron et al. 2022), and is a conservative option in terms of propagating uncertainty via within-individual variability.

*Literature Cited*

Cameron D, Hartig F, Minnuno F, Oberpriller J, Reineking B, Van Oijen M, Dietze M. Issues in calibrating models with multiple unbalanced constraints: the significance of systematic model and data errors. 2022. Meth Ecol Evol;13(12)2757-2770.

James G, Witten D, Hastie T, Tibshirani R. An Introduction to Statistical Learning with Applications in R. 2017. Spring Science and Business Media.

Moran P. A test for the serial independence of residuals. 1950. Biometrika;37-178-181.

Thum T, MacBean N, Peylin P, Bacour C, Santaren D, Longdoz B, Loustau D, Ciais P. The potential benefit of using forest biomass data in addition to carbon and water flux measurements to constrain ecosystem model parameters: Case studies at two temperate forest sites. 2017. Ag For Met;234:48-65.

Wood SN. Fast stable restricted maximum likelihood and marginal likelihood estimation of semiparametric generalized linear models. 2011. J. Roy Stat Soc;73(1):3-36.

Wood SN. Generalized Additive Models: An Introduction with R. 2017. Chapman and Hall.

Wright MN, Ziegler A. Ranger: A fast implementation of random forests for high dimensional data in C++ and R. 2017. J Stat Soft;77(1):1-17.
