# Supplementary material for: Chinook salmon depth distributions on the continental shelf are shaped by interactions between location, season, and individual condition
Source: Mov Ecol. 2024 Mar 15;12:21. doi: 10.1186/s40462-024-00464-y (PMC11337652; doi:10.1186/s40462-024-00464-y)
Supplement: Supplementary file 3 — Additional file 3. Supplementary figures. [file 40462_2024_464_MOESM3_ESM.pdf]

Supplementary Figures

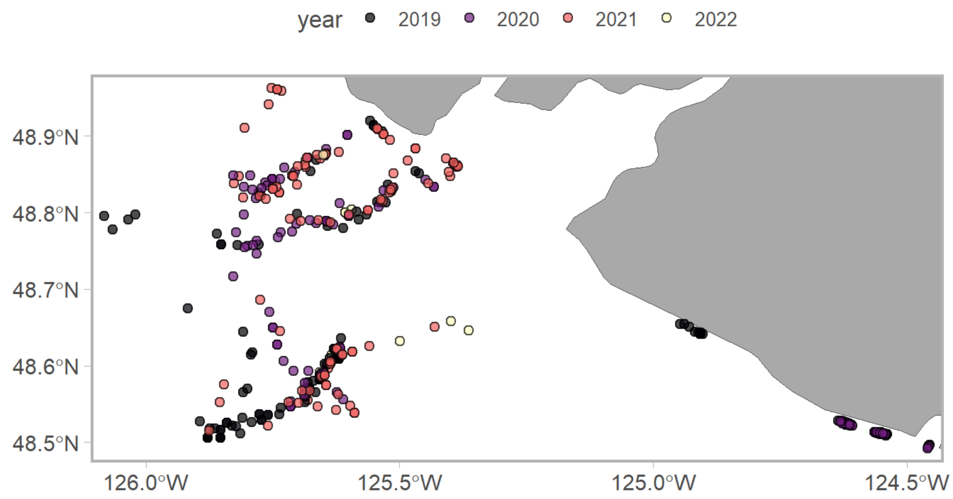

Figure S1: Locations of tag releases. See Figure 1 for additional information.

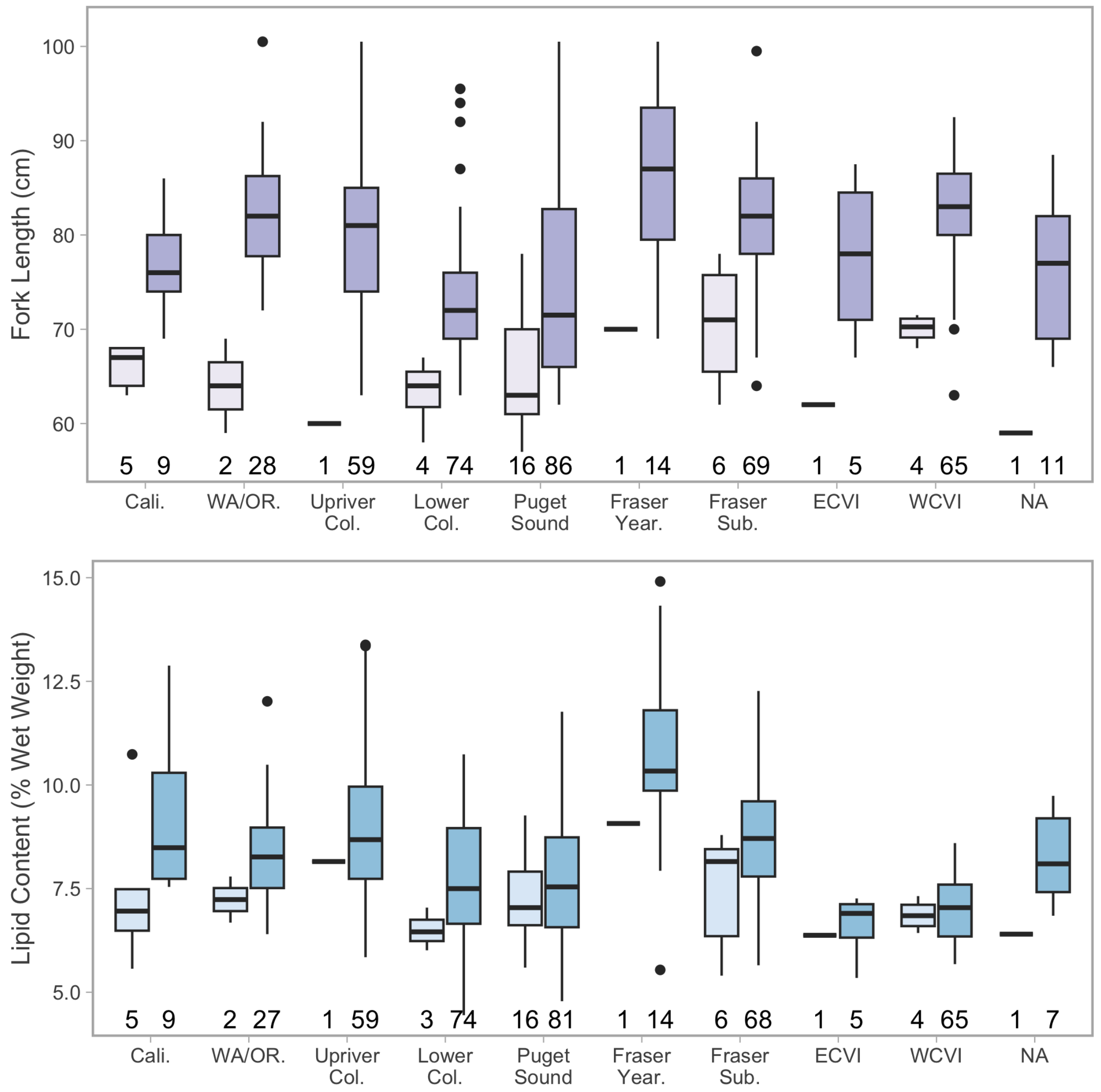

Figure S2: Chinook salmon fork length (top) and estimated whole body lipid content (bottom). Immature (mature) individuals are represented by lighter (darker) colours. Stock aggregate- and stage-specific sample sizes are shown as numerics under each boxplot. Here we assumed individuals with a maturation probability less than 0.5 were immature and greater than 0.5 were mature. Stock aggregate abbreviations and contributing stocks are defined in Table S1.

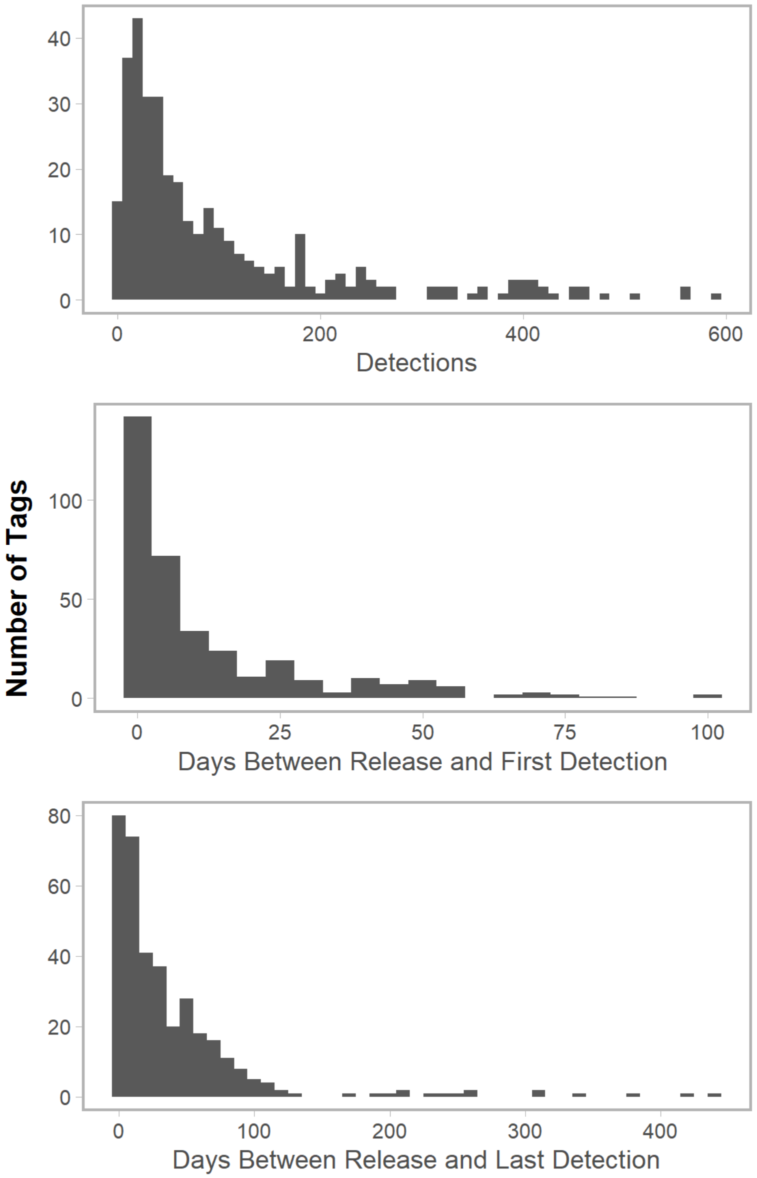

Figure S3: Number of detections (top), number of days between release and first detection (middle), and number of days between release and last detection (bottom) per tagged Chinook salmon. We excluded tags with the highest number of detections (greater than 600 (n = 17); top row) and with the longest period prior to detection (greater than 100 days (n = 16); middle row).

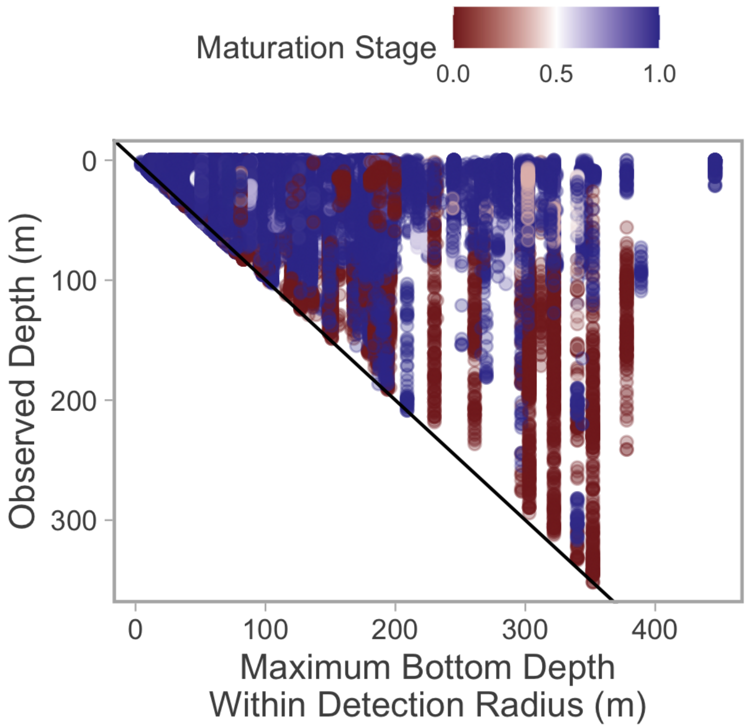

Figure S4: Observed Chinook salmon depth relative to bottom depth (maximum within a receiver's detection radius). Black line represents the ocean bottom.

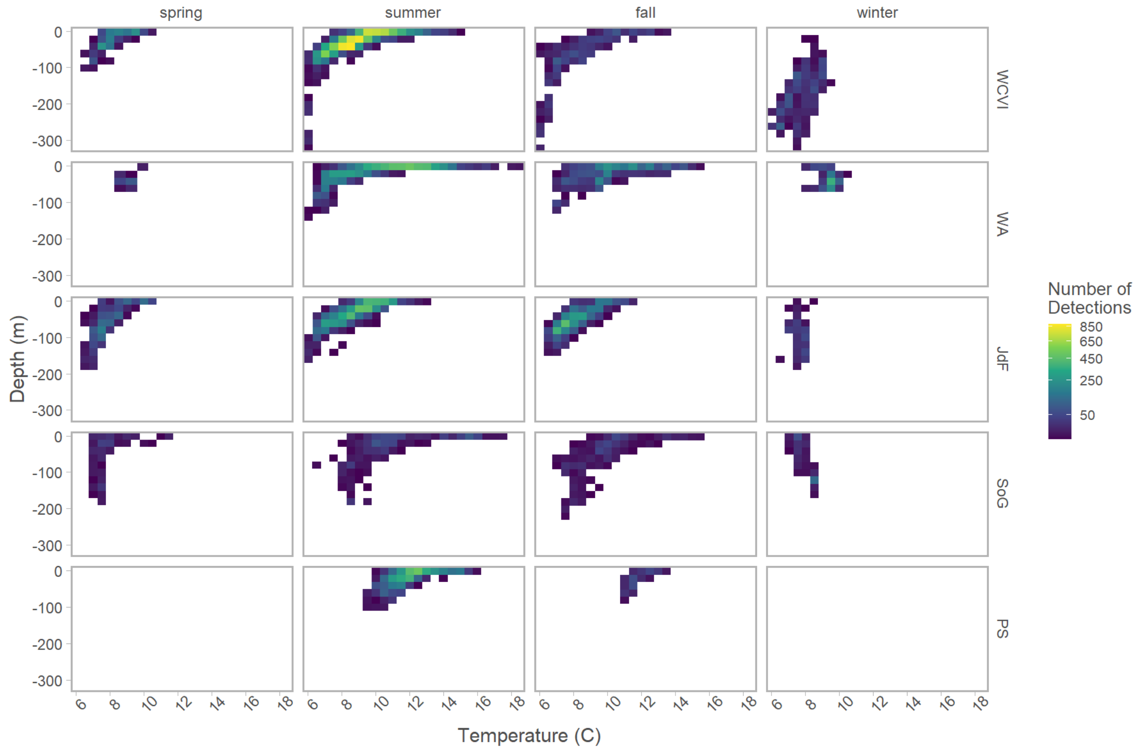

Figure S5: Seasonal and spatial variation in temperature-at-depth coincident with Chinook salmon detections. Seasons defined as: winter (December-February), spring (March-May), summer (June-August), and fall (September-November). To allow for interpretability we grouped receivers into the following spatial regions: westcoast Vancouver Island (WCVI), coastal Washington (WA), Juan de Fuca Strait (JdF), Strait of Georgia (SoG), and Puget Sound (PS).

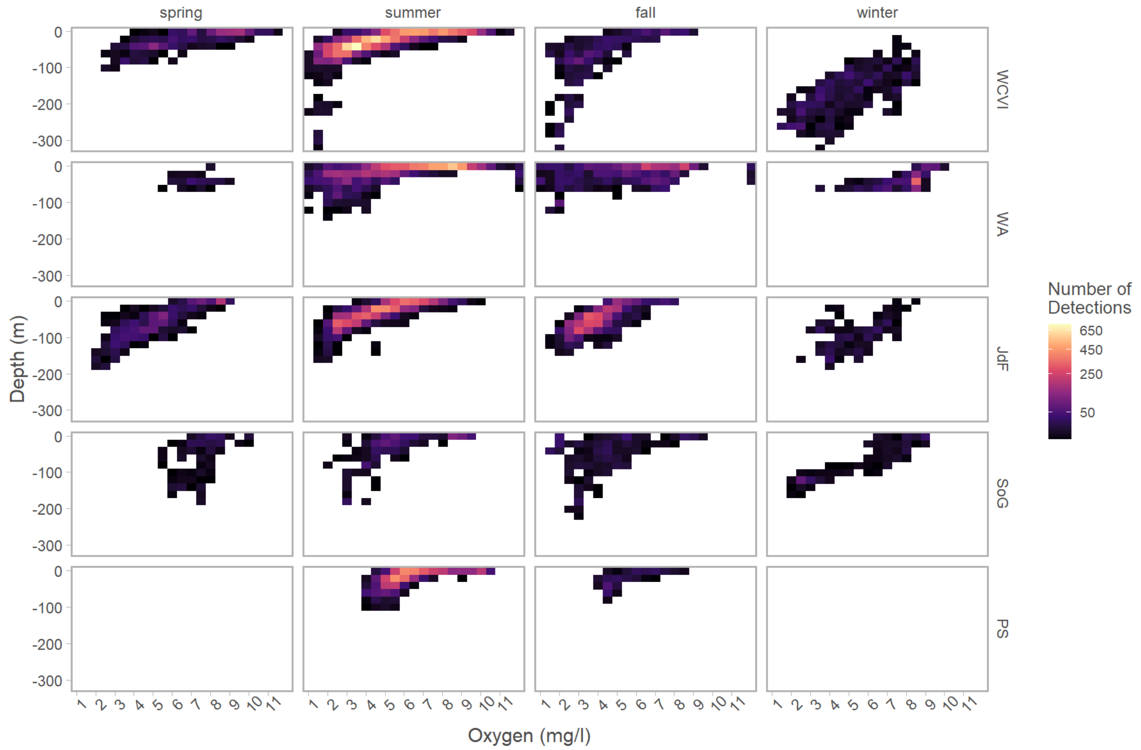

Figure S6: Seasonal and spatial variation in dissolved oxygen concentration (mg/l) coincident with Chinook salmon detections. Seasons defined as: winter (December-February), spring (March-May), summer (June-August), and fall (September-November). To allow for interpretability we grouped receivers into the following spatial regions: westcoast Vancouver Island (WCVI), coastal Washington (WA), Juan de Fuca Strait (JdF), Strait of Georgia (SoG), and Puget Sound (PS).

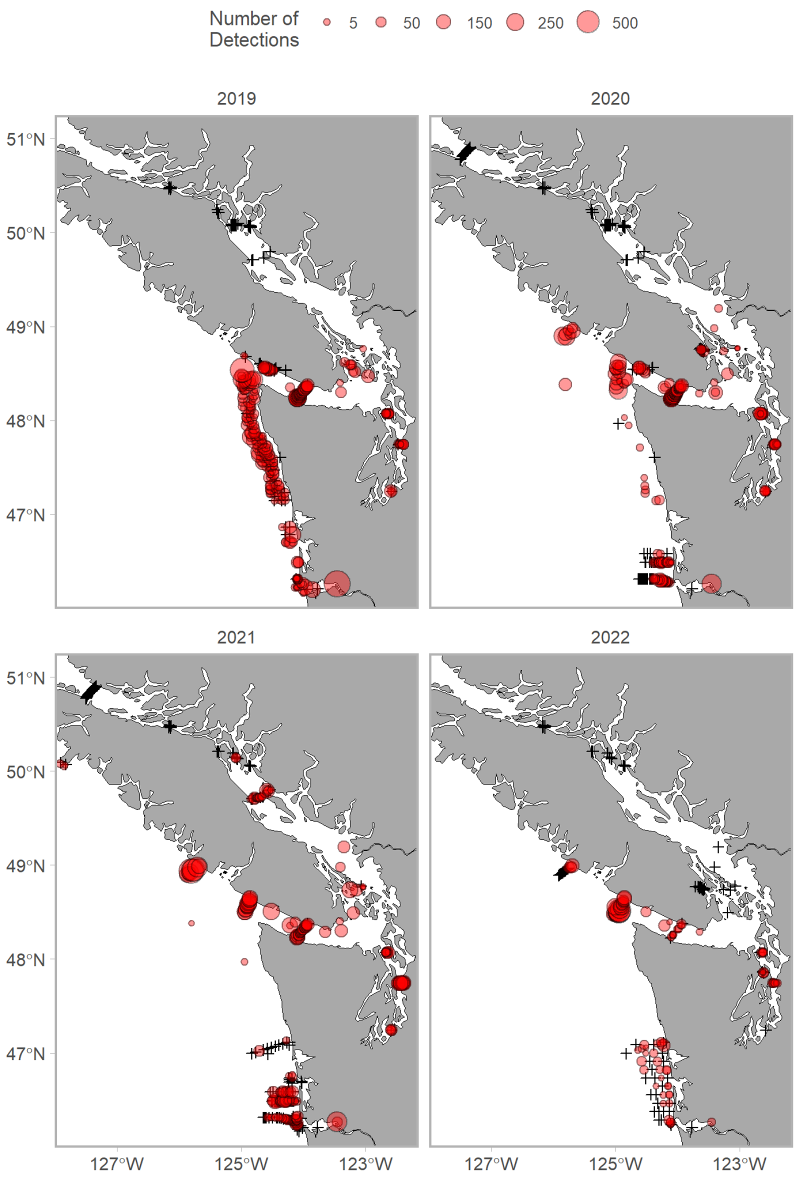

Figure S7: Number of individual Chinook salmon detections at each acoustic receiver. Locations that were not used in model fitting (no ROMS data available or no detections) are shown as crosses.

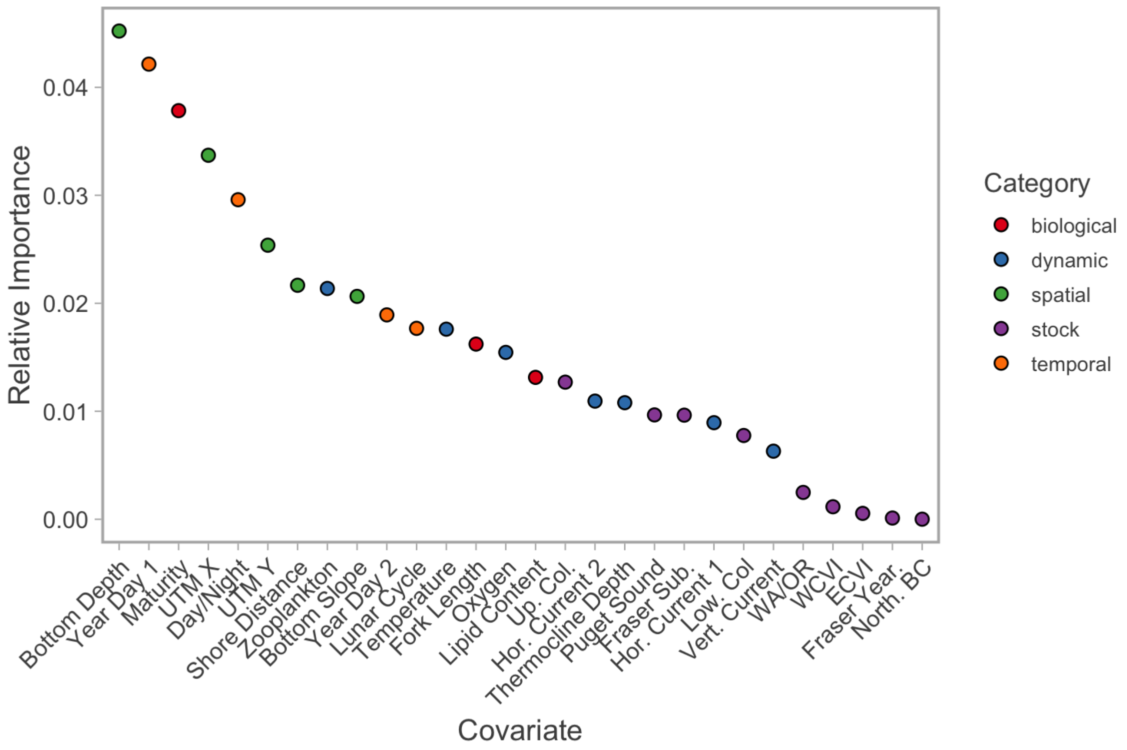

Figure S8: Relative importance of explanatory variables in random forest regression model including stock aggregate identity (equivalent to Figure 3). Importance is quantified as the difference (mean among trees) in root mean square error when a given variable is excluded during out-of-bag prediction. An uncorrelated random variable will have a difference in root mean square error of zero. Colors represent categories of explanatory variables (note the purple 'stock' categories at the right).

Supplementary Methods

Detections Data and Preprocessing

It was not feasible to conduct a comprehensive range test given the spatial scope and seasonal variation in environmental conditions; however, 800 m is a conservative estimate of the detection radius of tags used here [81]. Receivers record the unique tag code, a detection timestamp, and an estimate of depth (precision +/- 10 m). Detections and receiver metadata were either provided directly by the scientists managing the arrays or by the Ocean Tracking Network (https://oceantrackingnetwork.org/). Prior to statistical analyses, we removed false detections using the glatos R package ($< 1\%$) of detections assuming a short interval criteria of 3600 seconds [82] or more than 2.5 m below the maximum bottom depth within a receiver's detection radius. We fixed depth estimates less than 2.5 m below the maximum bottom depth ($< 1.0\%$ of detections) at 0.5 m above the maximum bottom depth value and estimates less than 0.1 m below the surface (i.e., at surface or slightly above surface) at 0.1 m below the surface to allow for logit transformations. A small number of tag transmissions (0.005%) were detected by multiple receivers simultaneously. In these cases we used the among receiver mean for each explanatory variables when fitting models.

Seasonal Effects

We converted calendar day to two composite variables that cumulatively represent a cyclical process,

$$d_1 = \cos\left(2\pi\frac{d}{365}\right), \tag{1a}$$

$$d_2 = \sin\left(2\pi\frac{d}{365}\right), \tag{1b}$$

where d is calendar day. We note that the use of composite variables involves trade-offs. When a single attribute (i.e., calendar day) is split into two covariates, its weight

relative to other covariates is inflated. Additionally the split rules associated with machine learning algorithms do not process linked covariates simultaneously, resulting in a subset of regression trees containing d_1, but not d_2, or vice versa. Nevertheless, we decided that an imperfect cyclical representation was better than alternative methods of incorporating seasonal variability that would assume independent or linear relationships through time.

LiveOcean ROMS Model

The LiveOcean implementation applies ROMS to a model domain that includes all of Oregon, Washington, the Vancouver Island coastal waters, and the Salish Sea. In the horizontal plane, the model grid follows lines of constant latitude and longitude, with higher resolution (500 m) in most of the Salish Sea and Washington coastal estuaries and lower resolution (up to 3 km) in the open ocean. The grid is terrain-following in the vertical direction, with 30 levels following the bathymetry and free surface, and with higher resolution near the sea surface and bed.

Model forcing includes open ocean boundary conditions from a global ocean model [83]; atmospheric forcing from a regional weather model [84]; tidal forcing; and daily averaged flow from 45 rivers. In addition, LiveOcean includes a nitrogen, phytoplankton, microzooplankton, detritus, and oxygen (NPZDO) biogeochemical model [85, 86].

LiveOcean is run in a data assimilative mode to generate daily forecasts, from which we extracted data at specified spatiotemporal coordinates (northing, easting, depth, date, and hour of tag detections) using linear interpolation routines in the xarray Python module [87].

Data Imputation

Before fitting models, we imputed missing values for stations where some, but not all, ROMS variables were missing due to boundary conditions (≈ 1-10% of observations) and several fish that were missing lipid content estimates (due to equipment malfunction; 2% of observations). Stations with no ROMS data were excluded. We used k-Nearest Neighbor Imputation, using the R package VIM [88], to impute all variables. ROMS variables were imputed based on spatial location, date, distance to shore, mean bottom depth, and mean slope. Full body lipid content was imputed based on fork length, stock identity (as inferred from genetic stock identification via single nucleotide polymorphisms [44]), maturity stage, and tagging year.

Life History Stage Model

The maturity stage at the time of tagging could not be inferred for a subset of individuals because they were not detected in terminal areas due to mortality or because detection efficiency in their natal system was very low. To predict the maturity stage of "unknown" individuals, we used data from 291 individuals where maturation stage at the time of tagging could be identified post-hoc based on detection histories.

Specifically we fit a hierarchical generalized linear model that modelled the probability of maturity using a Bernoulli distribution and a logit link:

$$y_i \sim \text{Bernoulli}(\mu_i), \tag{1}$$

$$\text{logit}(\mu_i) = \beta_d d_i + \beta_l l_i + \alpha_s, \tag{2}$$

$$\alpha_s \sim \text{Normal}\left(\mu_{\alpha_s}, \sigma^2_{\alpha_s}\right), \tag{3}$$

where y_i represents the binomial maturation stage at the time of tagging (0 immature, 1 mature) for individual i and μ is the mean probability of maturity. The β parameters

represent slope estimates for tagging date (year day) d and fork length l, while α_s represents a random intercept for stock identity.

We fit the model in a Bayesian framework with weakly informative priors (μ_{α_s} = Normal (0, 10), β = Normal (0, 2) and σ_{α_s} = Cauchy (0, 1)) in the R package brms [89]. The model was fit with four chains each with 1500 iterations, 750 of which were discarded as warmup. Posterior checks indicated the chains mixed well and all model parameters had an effective sample size greater than 900 and an $\hat{R}$ less than 1.01, suggesting convergence.

The probability of maturity at the time of tagging was correlated with tagging date and fork length ($\beta_d = -0.08$ and $\beta_l = 0.32$) with significant variability among populations ($\sigma_s = 1.63$). As a result, larger fish and those tagged early in the year were the most likely to mature in the year they were tagged Figure S9. The fitted model had an 87% correct classification accuracy, with a classification threshold of 50%, within the training dataset.

For individuals with unknown maturation stage, we used the median estimated posterior maturation probability as a continuous covariate in machine learning models. When reporting summary statistics related to mature and immature individuals (e.g., mean size and lipid content) we assumed individuals with a less than 50% probability of maturation were immature.

Machine Learning Model Tuning and Selection

To identify the framework with the best out-of-sample predictive performance we fit six alternative models within two machine learning frameworks (gradient boosting machines (GBM) and random forests (RF)) and three different response variable distributions (untransformed depth, depth scaled by maximum bottom bathymetry (bathymetric depth ratio), and logit transformed bathymetric depth ratio). For each of the six models we evaluated all combinations of hyperparameters via a grid search

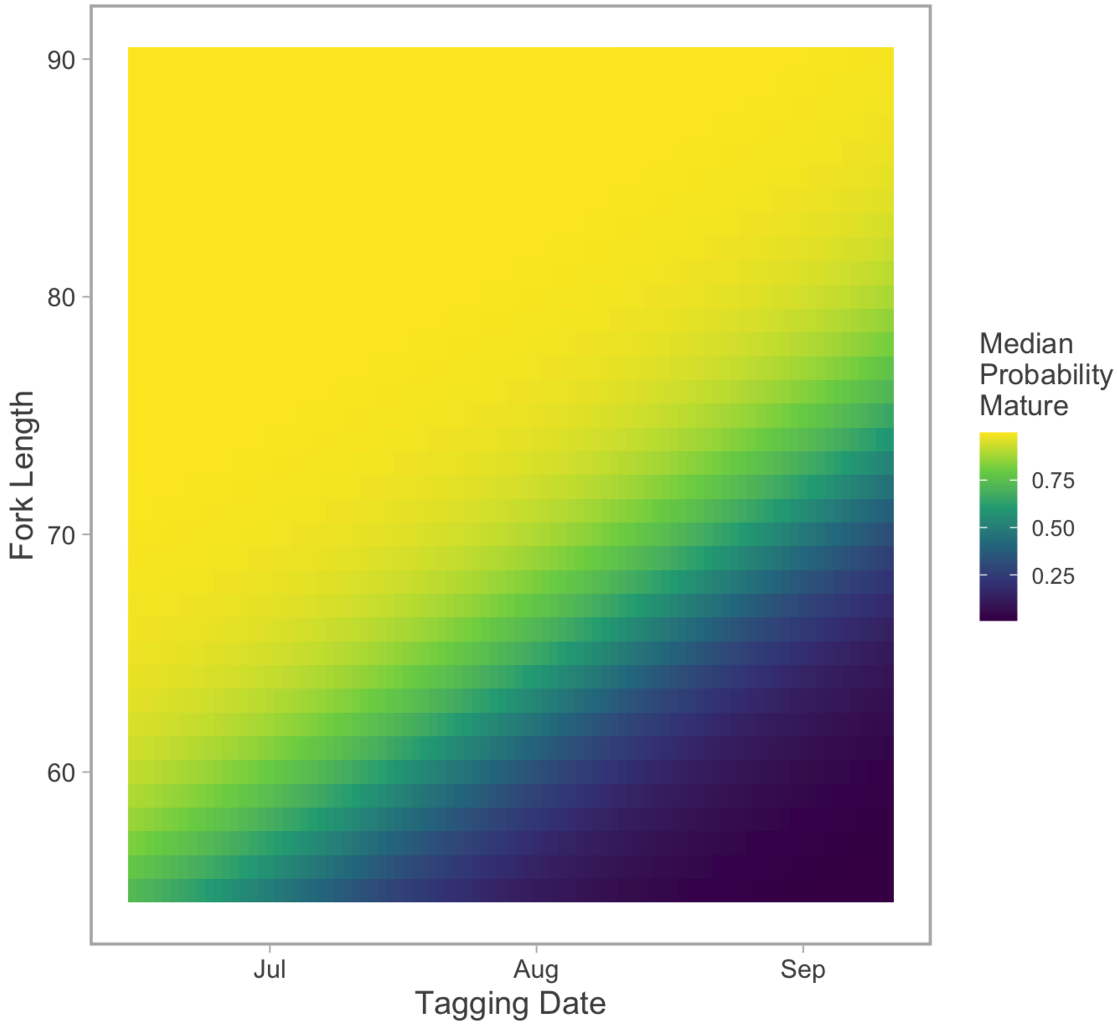

Figure S9: Median posterior predicted probability of maturity at time of tagging as a function of fork length and tagging date.

tuning procedure (Table S1) and selected the optimal suite of hyperparameters based on root mean square error (RMSE).

We next fit each model, fixing hyperparameters at their respective optimal values (lowest RMSE; Figures S10, S11), to both the training and out-of-sample testing datasets (described in methods above), then calculated the RMSE of predictions. In the case of transformed response variables (bathymetric depth ratio and logit-scaled bathymetric depth ratio), we backtransformed predictions and calculated RMSE relative to untransformed observations.

Table S1: Model hyperparameters.

Model	Hyperparameter	Values
Gradient Boosting Machine	Interaction Depth	2, 5, 10
	Number of Trees	10, 20, 50, 100, 150, 200, 250, 300
	Shrinkage	0.01, 0.1
	Minimum Number of Observations in a Node	5, 10, 20
Random Forest	Number of Parameters at Split	2, 3, 5, 7, 9, 11, 13, 15, 17
	Split Rule	variance, extratrees (extremely random trees)
	Number of Trees	1000, 1500, 2000, 2500, 3000

Random forest models tended to outperform gradient boosting machines, though these differences were relatively modest when predictions were evaluated using held-out testing data. The relative depth random forest model had the best performance with training and held-out testing data (Figure S12). Therefore we selected a random forest model fit to bathymetric depth ratio, with 1000 trees, and that evaluated 17 variables at each split for our primary analysis (Figure S12).

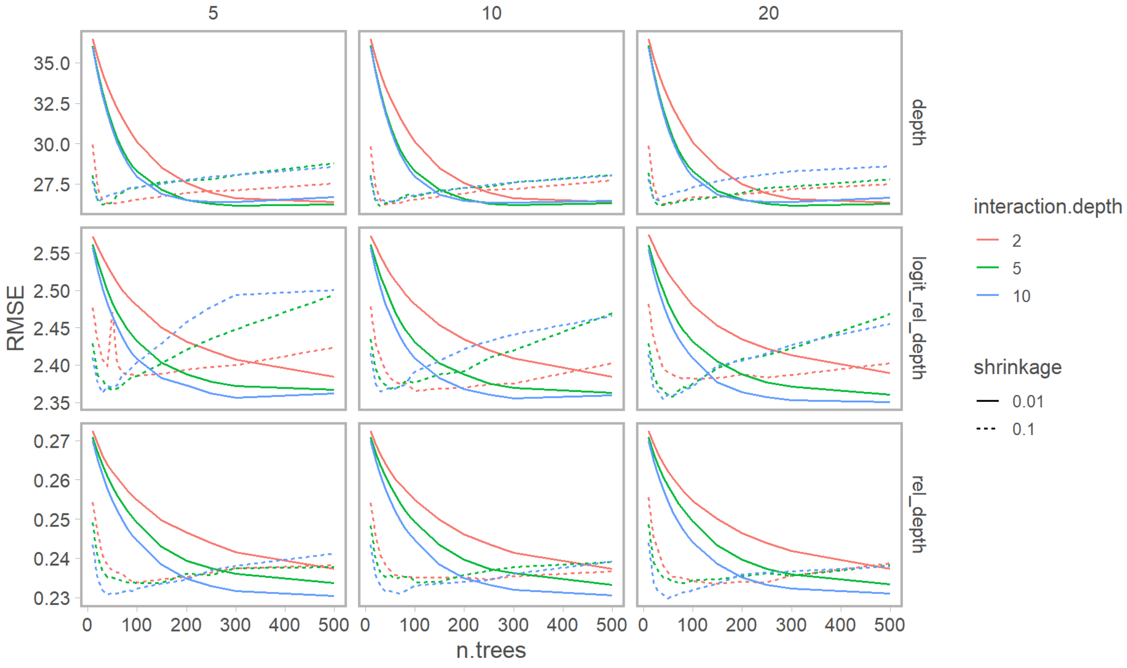

Figure S10: Performance of alternative gradient boosting machine model hyper-paramters fit to training data. The columns represent different minimum observations per node. Root mean square error was calculated in transformed space (i.e., bathymetric depth ratio and logit-scaled bathymetric depth ratio were not back-transformed), so difference in scale among response variable types differs (figure rows).

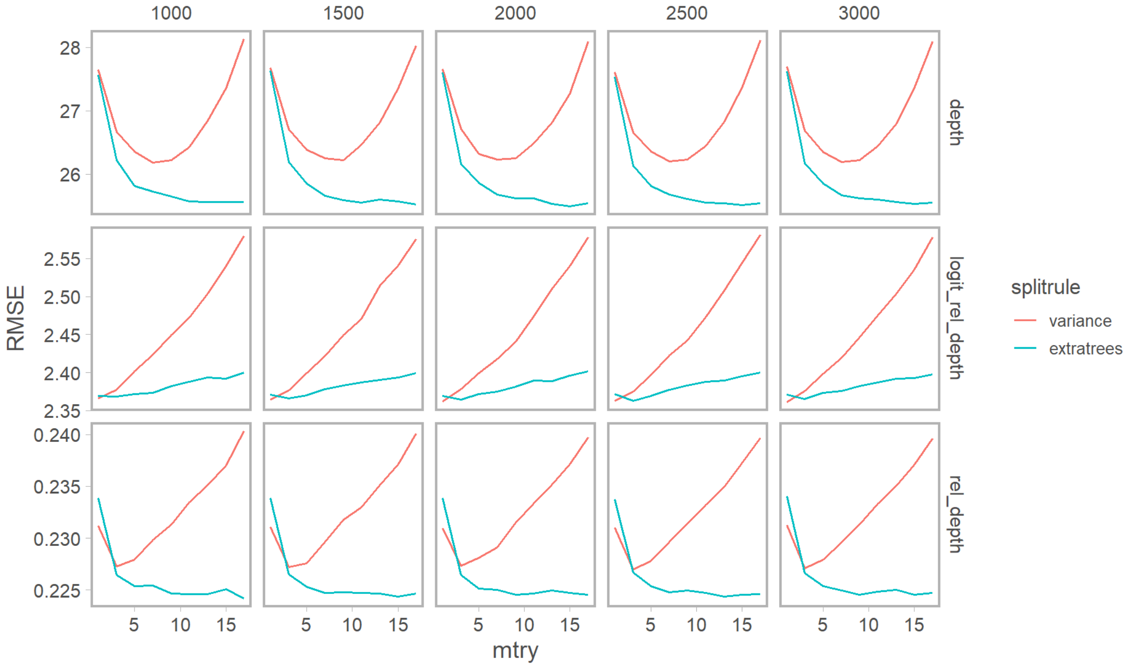

Figure S11: Performance of alternative random forest model hyperparamters fit to training data. Root mean square error was calculated in transformed space (i.e., bathymetric depth ratio and logit-scaled bathymetric depth ratio were not back-transformed), so difference in scale among response variable types differs (figure rows).

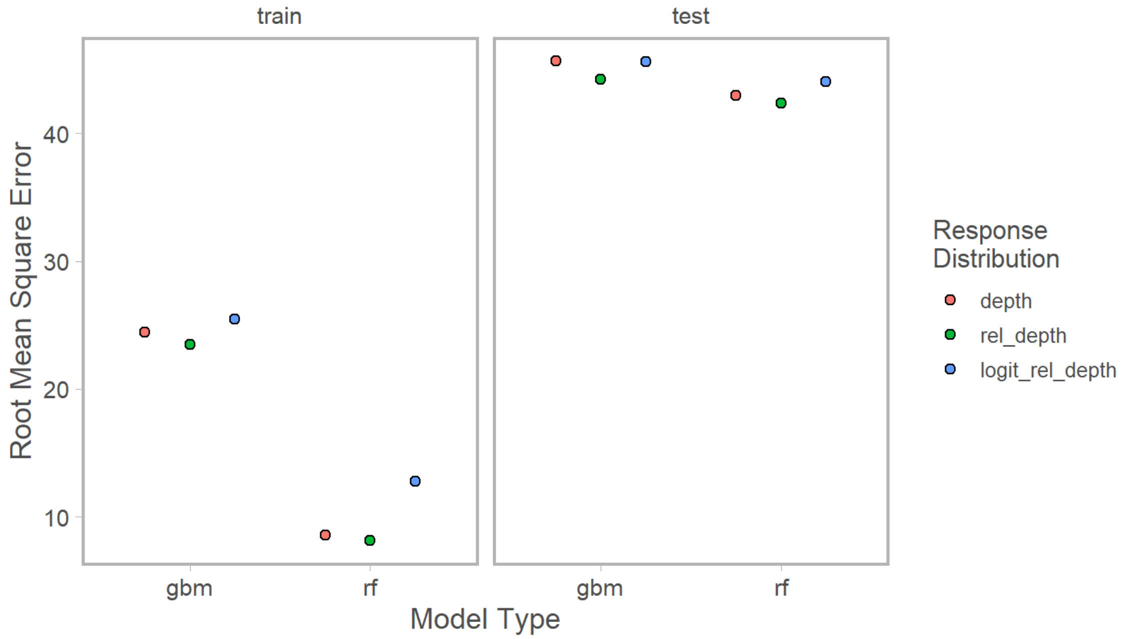

Figure S12: Predictive performance of six candidate machine learning models relative to testing (left) and out-of-sample training data (right). Root mean square error was calculated in real space (i.e., bathymetric depth ratio and logit-scaled bathymetric depth ratio were back-transformed).
